# A switch from α‐helical to β‐strand conformation during co‐translational protein folding

**DOI:** 10.15252/embj.2021109175

**Published:** 2022-01-07

**Authors:** Xabier Agirrezabala, Ekaterina Samatova, Meline Macher, Marija Liutkute, Manisankar Maiti, David Gil‐Carton, Jiri Novacek, Mikel Valle, Marina V Rodnina

**Affiliations:** ^1^ CIC bioGUNE Basque Research and Technology Alliance (BRTA) Derio Spain; ^2^ Department of Physical Biochemistry Max Planck Institute for Biophysical Chemistry Gottingen Germany; ^3^ IKERBASQUE Basque Foundation for Science Bilbao Spain; ^4^ CEITEC Masaryk University Brno Czech Republic; ^5^ Present address: BREM (Basque Resource for Electron Microscopy) BIOFISIKA Institute (UPV/EHU‐CSIC) Leioa Spain

**Keywords:** cotranslational folding, nascent chain, ribosome, Translation & Protein Quality

## Abstract

Cellular proteins begin to fold as they emerge from the ribosome. The folding landscape of nascent chains is not only shaped by their amino acid sequence but also by the interactions with the ribosome. Here, we combine biophysical methods with cryo‐EM structure determination to show that folding of a β‐barrel protein begins with formation of a dynamic α‐helix inside the ribosome. As the growing peptide reaches the end of the tunnel, the N‐terminal part of the nascent chain refolds to a β‐hairpin structure that remains dynamic until its release from the ribosome. Contacts with the ribosome and structure of the peptidyl transferase center depend on nascent chain conformation. These results indicate that proteins may start out as α‐helices inside the tunnel and switch into their native folds only as they emerge from the ribosome. Moreover, the correlation of nascent chain conformations with reorientation of key residues of the ribosomal peptidyl‐transferase center suggest that protein folding could modulate ribosome activity.

## Graphical abstract

Co‐translational folding of a small β‐structured protein involves formation of a highly dynamic α‐helix inside the ribosome exit tunnel.

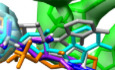

## Introduction

Understanding how proteins fold is of fundamental importance, as functional proteins are required for essentially every process of life. Proteins begin to fold on the ribosome during ongoing translation (Balchin *et al*, 2016; Cassaignau *et al*, 2020; Liutkute *et al*, 2020b). Secondary structure elements and small domains can form inside the exit tunnel of the ribosome, which provides a confined space enclosing at least 30 amino acids of the polypeptide nascent chain. The vectorial emergence of the nascent peptide and the pace of translation are important, as changing the order in which N‐ and C‐terminal parts of the protein emerge from the ribosome (Komar & Jaenicke, 1995; Marsden *et al*, 2018) or the local translation rates by synonymous codons in the mRNA (Clarke & Clark, 2008; Zhang *et al*, 2009; Buhr *et al*, 2016; Walsh *et al*, 2020) lead to increased misfolding and aggregation. The ribosome modulates folding by destabilizing the native structures and stabilizing intermediates that are either transient or not sampled upon folding in solution (Clark & King, 2001; Woolhead *et al*, 2004; Lu & Deutsch, 2005; Bhushan *et al*, 2010; Kaiser *et al*, 2011; Holtkamp *et al*, 2015; Liutkute *et al*, 2020a).

The detailed pathway of cotranslational folding is known for only a handful of proteins. Folding of α‐helical proteins often begins by forming α‐helical elements inside the exit tunnel (Woolhead *et al*, 2004; Lu & Deutsch, 2005; Bhushan *et al*, 2010), which are further compacted as the nascent chains grows longer (Holtkamp *et al*, 2015; Nilsson *et al*, 2015; Mercier & Rodnina, 2018). For example, folding of the α‐helical N‐terminal domain of protein HemK starts inside the exit tunnel by sequential docking of consecutive α‐helices, which compact into a stable native fold upon emerging from the ribosome (Holtkamp *et al*, 2015; Mercier & Rodnina, 2018; Liutkute *et al*, 2020a). Folding of β‐stranded proteins is less well understood. Several examples suggest that β‐structures can fold when parts of the nascent polypeptide emerge from the ribosome before the whole protein is released (Evans *et al*, 2008; Eichmann *et al*, 2010; Kelkar *et al*, 2012; Buhr *et al*, 2016; Cabrita *et al*, 2016; Guinn *et al*, 2018; Marsden *et al*, 2018; Notari *et al*, 2018). NMR studies suggested that nascent polypeptides remain unstructured inside the exit tunnel (Eichmann *et al*, 2010; Cabrita *et al*, 2016). However, small β‐structures, such as β‐hairpins, can fold inside the ribosome (Kosolapov & Deutsch, 2009; Marino *et al*, 2016). The exact conformation of such nascent peptide elements is not known due to the lack of structural data and the information on their dynamics is completely missing. For a specific case of β‐helix proteins, native‐like packing starts with a minimum set of four β‐helix coils at the tunnel exit port and continues sequentially as the nascent peptide emerges from the ribosome (Evans *et al*, 2008; Notari *et al*, 2018); interestingly, early folding on the ribosome may entail intermediates that are not found upon refolding in solution (Clark & King, 2001). On the other hand, several recent studies of globular β‐structured proteins suggest that the same key set of residues govern the final transition to the native state upon emerging from the exit tunnel and off the ribosome (Guinn *et al*, 2018; Marsden *et al*, 2018; Tian *et al*, 2018), but the structure of the nascent peptide inside the exit tunnel, the folding landscape, and the timing of nascent chain compaction remain unclear. Here, we explore the cotranslational folding pathway of a β‐structured protein from the earliest events inside the tunnel to its emergence as full‐length protein from the ribosome, using a combination of biochemical, biophysical, and structural techniques.

## Results

### Early events of CspA folding inside the ribosome

**Figure 1:**
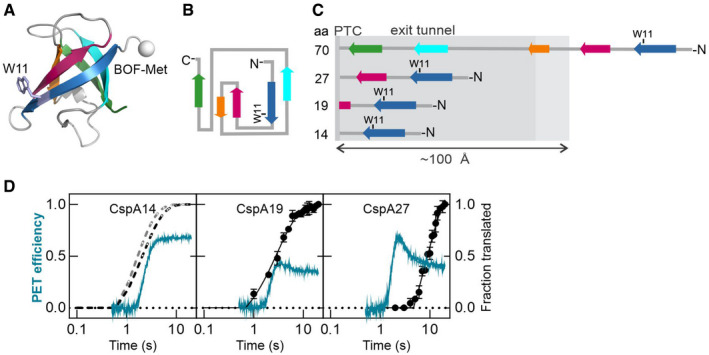
Cotranslational folding of CspA inside the polypeptide exit tunnel of the ribosome Structure of CspA from *E. coli* (PDB: 1mjc (Schindelin *et al*, 1994)). Strands 1–5 are colored blue, purple, orange, cyan, and green, respectively. Positions of the fluorescence reporter BOF and of W11 used as an intramolecular quencher are indicated.Topology diagram of CspA. Colors are as in A.Schematic of CspA nascent chain inside the exit tunnel. The area from the PTC to the exit tunnel vestibule is shown in light gray.Cotranslational compaction of CspA nascent chain inside the tunnel. PET efficiency (cyan) and translation (black) of 14, 19, or 27 aa‐long nascent chains as indicated. The average rate of translation for short nascent chains, 5.2 aa/s, was calculated from the translation kinetics for CspA19 (closed symbols) (Appendix Table S1) and used to estimate translation kinetics for CspA11 (W11 incorporation into peptide; gray dashed line) and CspA14 (black dashed line) (Method Details). PET time courses are averages of 5–6 technical and three biological replicates (*N* = 3). Translation time courses are averages with standard deviation of three biological replicates (*N* = 3). Source data are available online for this figure.

As a model protein, we have chosen the cold shock protein A (CspA) from *Escherichia coli*, which is a major regulator of the transcriptional response during cold adaptation in many bacteria. CspA is a β‐barrel protein (Fig 1A). It consists of 70 amino acids (aa) that form five β‐strands connected by loops. Strands 1–3 form an antiparallel β‐sheet that is closed into a barrel by packing strands 4 and 5 to strands 1 and 3, respectively (Fig 1B). In solution, CspA folds rapidly (within <5 ms) by a two‐state concerted mechanism (Reid *et al*, 1998; Vu *et al*, 2001). On the ribosome, strands 1–3 (aa 1–34) could form a β‐sheet upon emerging from the exit tunnel, whereas docking of strands 4 and 5 (aa 49–70) would only be possible after the release of the nascent chain from the ribosome (Fig 1C). To monitor the timing of folding during on‐going translation we used photoinduced electron transfer (PET) approach. Because PET relies on a fluorescence quenching effect of tryptophan (Trp) when in van der Waals distance of a suitable fluorescence reporter, this approach can be used to follow the compaction of the protein when the reporters that are distant in the linear polypeptide chain come close upon folding. We introduced Bodipy‐FL (BOF) at the N‐terminal Met of the nascent chain (Holtkamp *et al*, 2015; Liutkute *et al*, 2020a). As PET quencher, we utilized the native Trp residue at position 11 (W11). Because quenching is strongly distance‐dependent, PET signal between BOF‐Met1 and W11 in an extended polypeptide conformation is unlikely and can only occur upon dynamic compaction of the nascent chain. As a control for intrachain PET, as opposed to quenching by the ribosome which also contains potential quenchers, we used a Trp‐less CspA variant with the substitution W11L. Translation and folding of the modified variants was unchanged compared to the wild‐type CspA (Appendix Fig S1A–D). To investigate potential folding events inside the tunnel, we first used short constructs coding for the N‐terminal part of the protein (Fig 1C, Appendix Fig S1A). Upon translation of the CspA mRNA coding for the first 14 aa, we observe a clear PET signal, indicating that the nascent chain adopts a compact state inside the exit tunnel (Fig 1D, Appendix Fig S2A). Comparison of the translation rate (~5.2 aa/s) (Appendix Table S1) and the PET kinetics (Appendix Table S2) (lifetime τ = 2.5 s) suggests that the compaction occurs when the polypeptide chain incorporates 13 aa. Upon addition of the next 5 aa of the CspA nascent chain, the PET signal decreases slightly due to a rapid rearrangement that occurs as the chain grows (Fig 1D). Increase of the chain length to 27 aa results in a biphasic PET change with an initial increase followed by a decrease (Fig 1D). Both PET phases occur faster than the synthesis of the 27‐aa translation product. The high‐PET intermediate transiently accumulates due to a translational pause at about aa 19 (Appendix Fig S1A) and rearranges into a less compact stable state with further translation. Thus, a β‐stranded protein starts to compact inside of the exit tunnel and undergoes several conformational rearrangements during ongoing translation before emerging from the ribosome.

### CspA forms a dynamic α‐helix inside the ribosome

**Figure 2:**
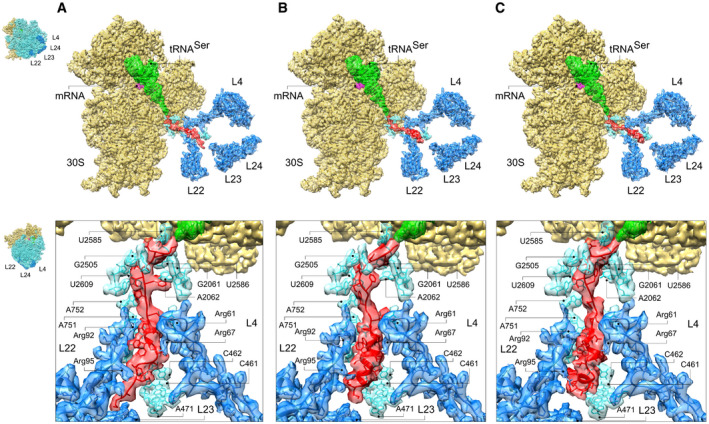
Cryo‐EM structures of CspA27 inside the exit tunnel Structure and nascent chain contacts in the exit tunnel for CspA27‐1.Structure and nascent chain contacts in the exit tunnel for CspA27‐2.Structure and nascent chain contacts in the exit tunnel for CspA27‐3. Data information: The cryo‐EM density is presented in transparent red (peptide), green (tRNA), violet (mRNA), yellow (30S subunit), and cyan/blue (the 50S subunit 23S rRNA and proteins, respectively). The rest of the 50S subunit (insets, blue) is removed for clarity. Insets show the orientation of the ribosome for each panel.

To explore the nature of CspA intermediates inside the exit tunnel, we determined the structure of the ribosome with the 27 aa‐long CspA nascent chain (CspA27) by cryo‐EM (Methods). After *in‐silico* particle sorting (Appendix Fig S3 and Appendix Table S3), we solved three structures that showed different conformations of the nascent chain and carried the P‐site tRNA^Ser^ corresponding to the mRNA codon 27. The resulting maps show strong density for the nascent chain, albeit at lower resolution than the surrounding ribosome elements due to its intrinsic dynamics. Atomic models were built to interpret the data, but because the medium resolution features of the nascent chains (4–6 Å) do not permit unequivocal assignment of the chain register, we interpreted peptide features in terms of secondary structure elements only. The backbone of the nascent chain is sufficiently well defined to represent snapshots of CspA folding inside the exit tunnel (Fig 2, Appendix Figs S4–S7, Movies [Link], [Link]).

The cryo‐EM densities for the CspA‐27 complexes reveal three different nascent chain conformations inside the tunnel (Fig 2). In the most prevalent conformation 2 (CspA27‐2), the nascent chain forms a compact α‐helix starting at the N‐terminus and up to the constriction formed by ribosomal proteins L4 and L22 (Fig 2B). In conformation 1 (CspA27‐1), the nascent chain is more extended at the N‐terminal part; the region immediately below the constriction site appears more compact (Fig 2A). In conformation 3 (CspA27‐3), which is the least populated, helix geometry in the central part of the nascent chain is disrupted in the middle helical turn region, which leads to the formation of two smaller compact modules (Fig 2C). This metastable conformation may represent a partially folded, distorted variant of the α‐helical conformation observed in CspA27‐2.

In all three conformations, the CspA nascent chain makes numerous contacts with exit tunnel residues. These interactions are clearly defined as the electron density corresponding to nearly every residue in the tunnel is very well resolved (Appendix Fig S6). Interactions involve conserved nucleotides U2585 and G2061 in the upper tunnel part close to the PTC; the contact to G2061 is absent in CspA27‐3 (Fig 2). In all conformations, U2609 and A2062 are in contact distance with the nascent chain. Right after the constriction formed by L4 and L22, nucleotide A751 interacts with the helical part of the chain; the contact seems slightly altered in CspA27‐3. Finally, the kink observed in CspA27‐3 creates a flexible point within the structure that allows rocking of the distal end, which leads to the formation of a set of interactions between the N‐terminal part of the nascent chain and 23S rRNA nucleotides 460–462; these contacts are not observed in the other conformations.

While most of the 23S rRNA elements lining the tunnel wall have similar local conformation in the three structures, the positively charged Arg residues of proteins L4 and L22 move to adjust to the nascent chain conformation (Movies EV4 and EV5). Arg61 of L4 has identical orientation in CspA27‐1 and ‐2, but contacts the nascent chain only in CspA27‐1. In CspA27‐3, Arg61 adopts a different conformation, but still contacts the nascent chain. L22 residues Arg92 and Arg95 have different orientations and contact different positions of the nascent peptide in compact and extended conformations. While the contact of Arg92 with the nascent chain is preserved in CspA27‐3, Arg95 is more flexible and makes no contact with the nascent chain due to the reorientation of the N‐terminal helical module.

### Late folding events on the ribosome

**Figure 3:**
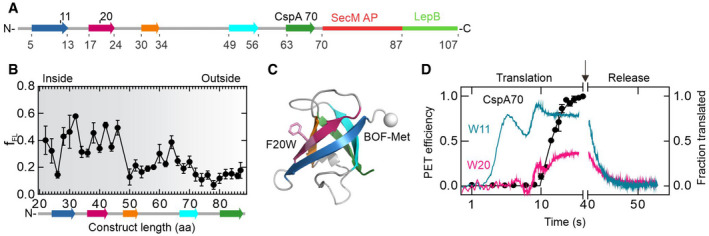
Folding transitions upon synthesis of full‐length CspA70 Schematic of the FPA sensor. CspA strands 1–5 are colored as in Fig 1, followed by SecM arrest peptide (red), and a fragment of LepB (green).Force profile of CspA folding. f_FL_ is the fraction of the full‐length product formed during *in vitro* translation; error bars indicate SEM calculated from three independent biological replicates (*N* = 3). The schematic underneath shows the sequence of CspA at the indicated aa chain length.Position of W20 in the structure of CspA.Cotranslational folding and release of CspA70 from the ribosome. PET efficiency was measured using W11 (cyan) or W20 (magenta) as quenchers; shown are averages of three biological replicates with 6 technical replicates each (*N* = 3). Translation of CspA70 (black); shown are averages with standard deviation of three biological replicates (*N* = 3). The arrow shows the time of release factor ArfB addition. Source data are available online for this figure.

Next, we used a high‐resolution force profile approach (FPA) to map the appearance of folding events. We generated CspA constructs of different lengths and added a SecM stalling sequence followed by a 23 aa‐long sequence of LepB, the latter serving as resumed translation readout (Fig 3A). A mechanical pulling force originating from nascent protein folding (Goldman *et al*, 2015; Kemp *et al*, 2020) can alleviate the SecM stalling and restart translation (Ismail *et al*, 2012; Goldman *et al*, 2015; Nilsson *et al*, 2015). This results in a full‐length peptide product that can be distinguished from the stalling product on SDS‐PAGE (Appendix Fig S2B). By calculating the fraction of the full‐length product, we identify high‐tension folding events with the CspA nascent chain inside the tunnel, which persist until the nascent chain has grown to 49 aa (Fig 3B). Upon moving further toward the tunnel exit, CspA undergoes a tension‐generating rearrangement at about 64 aa (Fig 3B), which indicates yet another structural rearrangement of the nascent chain.

To capture this late folding event during ongoing translation, we again used the PET approach, this time using full‐length CspA70 and Trp quenchers at positions 11 (W11) or 20 (W20) (Figs 3C and D, Appendix Fig S2A). With W11 as a quencher, we find a PET signature of CspA27 followed by further changes in PET efficiency, which occur cotranslationally with the synthesis of CspA70 (Fig 3D). PET from W20 also monitors this late rearrangement, albeit at lower efficiency. As W20 is far apart from the N‐terminal BOF in a linear nascent chain, it requires a dynamic compaction of the nascent chain to generate PET. The dynamic behavior of the nascent chain is further supported by PET‐FCS experiments, which indicate that dynamic quenching of the N‐terminal fluorescence reporter by W11 occurs in the sub‐µs to µs time range (Appendix Fig S2C and Table S4). Upon release of the nascent chain, PET efficiency decreases to zero due to the formation of a stable native conformation, which terminates the dynamic fluctuations of the N‐terminus towards W11 and W20 (Fig 3D).

### α‐helix rearranges into a β‐hairpin in the vestibule of the ribosome

**Figure 4:**
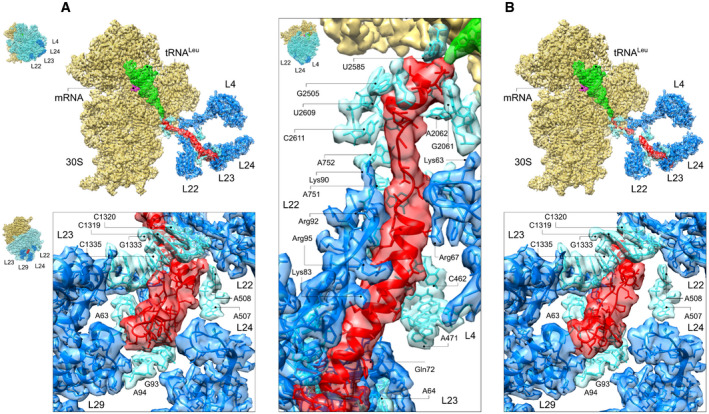
Conformation of the full‐length nascent CspA70 Overall structure and nascent chain interactions for conformations 1 (CspA70‐1). Upper panel, cut‐away view as in Fig 2. Lower panel shows close‐up view of the exit port. Panel to the right shows close‐up view of the central part of the tunnel.Overall structure and nascent chain interactions for conformations 2 (CspA70‐2). Upper panel, cut‐away view as in Fig 2. Lower panel shows close‐up views of the exit port. Data information: The cryo‐EM density is presented in transparent red (peptide), green (tRNA), violet (mRNA), yellow (30S subunit), and cyan/blue (the 50S subunit 23S rRNA and proteins, respectively). The rest of the 50S subunit (insets, blue) is removed for clarity. Insets show the orientation of the ribosome for each panel.

The cryo‐EM analysis shows the CspA70 nascent chain attached to tRNA^Leu^ at the PTC as it traverses the tunnel and emerges at the exit port. We obtained two structures of CspA70 that differ in the conformation of the nascent chain. Local resolution is generally lower due to broader conformational fluctuations, but similarly to the CspA27 complexes, the nucleotides and protein residues forming the ribosomal tunnel walls are well ordered (Fig 4A–C, Appendix Figs S8–S10 and Appendix Table S3, Movies EV6 and EV7). In the more stable conformation (CspA70‐1) the whole nascent chain backbone is traceable. The C‐terminal part of the nascent chain forms a short compact module within the contact range with PTC nucleotides G2061, A2062, G2505, U2585‐U2586 and, to a lesser extent, U2609 (Fig 4A). Below the L4‐L22 constriction site, the nascent chain forms a long helical rod of approximately 25 residues that extends toward the central part of the tunnel (Fig 4A, right panel). Residues A751 and A1614 of 23S rRNA contact the nascent chain in the upper part of the helical module, whereas several residues of the extended loop of L22 (Arg95, Arg92, Arg84, Lys83, Gly91, Ala93, and Ile85) pack against one face of the helix. In the lower part of the tunnel, the L23 β‐hairpin loop residues Arg69 and Gln72, and nucleotide A1321of 23S rRNA also interact with the nascent chain. At the opposite face, nucleotides A507–A508 establish interactions with the distal part of the helical density. Despite the extensive array of contacts, the peptide remains highly dynamic as evidenced by the presence of poorly resolved side chain densities. The spatial constraints imposed by the tunnel walls disfavor β‐structure formation; density that can accommodate a structured β‐hairpin is observed only upon reaching the vestibule, as more space for chain rearrangements becomes available. The structure is partially stabilized by the interactions with 23S rRNA nucleotides A63, G93‐U92, and C1335‐A1336, as well as by weak contacts with the protruding loops of L24 and L29 at the exit port.

In the second class of complexes, CspA70‐2 (Fig 4B), the density for the nascent chain is fragmented in the upper and central regions of the tunnel, and only when the peptide reaches the distal part, there is evidence for a more stable helical structure formation. The N‐terminal residues form a hairpin at the vestibule, which contacts 23S rRNA nucleotides and L29, whereas the interaction with the extended loop of L24 observed in CspA70‐1 is lost. Comparison of the two structures confirms that the nascent chain remains highly dynamic (Movie EV8), in agreement with the PET results (Fig 3D, Appendix Fig S2A–C). We note that the PET efficiency represents not only the conformational snapshots visualized by cryo‐EM, but rather an ensemble of structures some of which may be too scarcely populated to be captured by structural studies (Appendix Figs S3 and S8), but contribute to the high PET efficiency. The onset of the β‐hairpin formation likely explains the increased tension in the FPA profile at 64 aa (Fig 3B), which marks the timing of the conformational switch from the α‐helical structure to the β‐hairpin.

### Nascent chain conformation and ribosome activity

**Figure 5:**
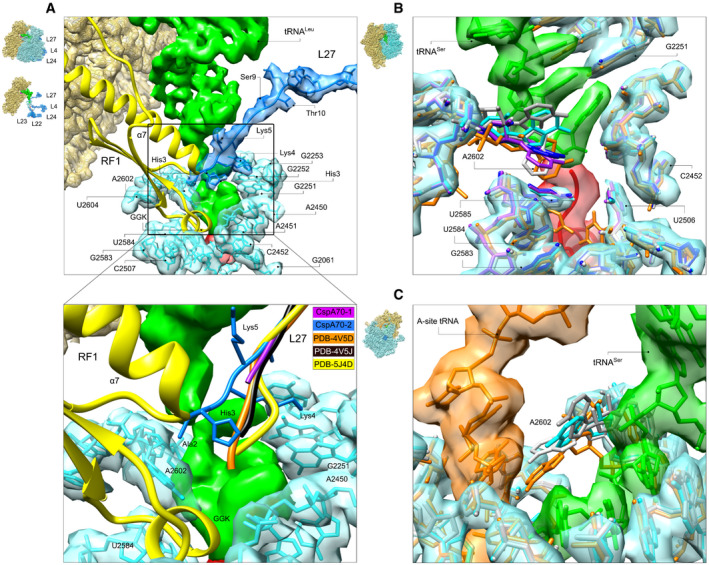
PTC conformation Docking of the catalytic GGQ motif into the PTC of CspA70‐2. The extended domain III of RF1 (PDB‐5J4D; Svidritskiy *et al*, 2016) containing the universally conserved GGQ loop and the extended *α*7 helix (yellow) is superimposed into the CspA70‐2 structure to highlight potential steric occlusion; stabilization of L27 N‐terminus by nucleotide A2602 would result in clash of RF1 extended *α*7 helix with residues Ala2 and His3 of L27. The L27‐2602 contact can be visualized when the map is contoured at around 1.7σ level. Note that the induced PTC structures of elongation and termination complexes are very similar except for A2602, which is buried in the pocket formed by RF1 domain III in the termination complexes, rather than projecting toward the A site as in the elongation complexes. A2602 position in the CspA70 conformations is closer to that shown in the termination complexes. Lower panel shows close‐up view of the PTC region. CspA70‐2 density for L27 is removed for clarity. Protein L27 from termination complexes PDB‐5J4D (Svidritskiy *et al*, 2016) (yellow), PDB‐4V5J (Jin *et al*, 2010) (black), elongation complex PDB‐4V5D (Voorhees *et al*, 2009) (orange), CspA70‐1 (purple), and CspA70‐2 (blue) are superimposed. The color scheme for comparison between the L27 structures is shown graphically at the top right of the close‐up view panel. The cryo‐EM density for CspA70‐2 is presented in red (peptide), green (tRNA), yellow (30S), and cyan/blue (the 50S subunit 23S rRNA and L27, respectively).Close‐up of superimposed PTC nucleotides for CspA27‐1 (white), CspA27‐2 (cyan), CspA27‐3 (grey), CspA70‐1 (purple), and CspA70‐2 (blue) with the induced 70S elongation complex (PDB‐4V5D (Voorhees *et al*, 2009); orange). The cryo‐EM density corresponding to CspA27‐2 is presented in transparent red (peptide), green (tRNA), and cyan (23S rRNA).Close‐up of superimposed PTC nucleotides for CspA27 complexes and A‐ and P‐site tRNA bearing induced elongation complex PDB‐4V5D (Voorhees *et al*, 2009) (orange); the A‐site tRNA from PDB 4v5d (also in orange) is docked into the PTC of CspA27‐2. Color code for cyoEM density as in B. The density corresponding to A2602 was computationally removed for clarity. Data information: The 23S rRNA was used as reference for structure alignment in all panels. Insets show the orientation of the ribosome for each panel.

Peptide bond formation involves nucleophilic attack of the α‐amino group of the incoming aminoacyl‐tRNA on the carbonyl carbon of the P‐site peptidyl‐tRNA. In bacteria, universally conserved 23S rRNA nucleotides U2506, U2584, U2585, and A2602 at the core of the PTC monitor the accurate placement of the substrates (Bashan *et al*, 2003; Youngman *et al*, 2004). Their repositioning changes the PTC conformation from inactive to active (Schmeing *et al*, 2005; Voorhees *et al*, 2009). Although the PTC mainly consists of 23S rRNA, in bacteria L27 can approach the catalytic core via its long flexible N‐terminal extension (Voorhees *et al*, 2009; Jin *et al*, 2010; Svidritskiy *et al*, 2016). In the CspA27 map, the N‐terminus is not well resolved; in contrast, in the CspA70 structures, the L27 N‐terminus is more defined (Fig 5A, Appendix Fig S11). Especially in CspA70‐2, we were able to model the conserved AHKK terminal motif (amino acids 2–6; fMet is removed in the mature protein; Wall *et al*, 2015). L27 residues Gly8, Gly7, and Ala6 contact tRNA^Leu^ nucleotides G1 and C2, and L27 Lys5 side chain interacts with the tRNA phosphate backbone of G1. The Lys4 side chain stacks into a pocket formed by 23S rRNA nucleotides G2251–G2253. The L27 N‐terminal motif is reoriented compared to other ribosome structures, ultimately allowing the N‐terminal Ala2 to contact A2602 of 23S rRNA. This interaction may favor a PTC conformation that might disfavor the binding of translation release factors (Fig 5A, lower panel). Thus, although L27 does not contribute significantly to the peptidyl‐transfer reaction (Maracci *et al*, 2015), it may control the termination efficiency and provide a sensor that links the folding of the nascent chain to its release into solution via the stabilization of its flexible N‐terminal tail by A2602.

Finally, when we extend the PTC comparison to CspA27, most of the PTC nucleotides superimpose between all structures, except A2602, which adopts a rather distinct orientation depending on the nascent chain conformation (Fig 5B, Appendix Fig S12). Because A2602 is conformationally the most flexible nucleotide of the PTC, it shows weaker density features in comparison to the surrounding PTC residues. However, when examined at lower threshold (contoured at ~1.5σ), nucleotide A2602 cryo‐EM density can be observed distinctly displaced toward the A site in the extended CspA27‐1. This position could potentially alter placement of the incoming aa‐tRNA (Fig 5C) and suggests a mechanism whereby the nascent polypeptide folding could locally attenuate the elongation rate by modulating aa‐tRNA accommodation.

## Discussion

Here, we report the determinants and temporal dynamics that drive the cotranslational folding of β‐barrel protein CspA using biophysical (PET and PET‐FCS), biochemical (rapid *in vitro* translation, FPA), and structural (cryo‐EM) methods. Our data represent snapshots of nascent chains sampling the conformational space and provide a sensitive readout of the folding process (summarized in Movies EV9 and EV10). We show that a cotranslationally folding β‐stranded protein undergoes a dramatic conformational switch from an α‐helical to a β‐stranded conformation depending on the nascent chain length and its position in the exit tunnel. Nascent chains remain dynamic as long as they are attached to the ribosome, and fold into their native conformation only after being released from the ribosome This example illustrates how interactions of nascent chains with the ribosome alter the folding trajectory of a β‐stranded protein from a rapid two‐state pathway to a complex translation‐dependent landscape that is not populated in solution. Such ribosome‐induced folding landscapes may be particularly important for proteins that do not refold spontaneously in solution (To *et al*, 2021) and must thus depend on the folding power of the ribosome and the chaperones.

Programmed stalling sequences can interfere with the peptidyl transferase activity of the ribosome by repositioning the active‐site residues (Wilson *et al*, 2016; Su *et al*, 2017). In our CspA structures, which represent ordinary nascent chains that do not induce programmed stalling, different conformations of the nascent peptide are supported by a flexible network of interactions with the tunnel wall residues. This network extends toward the PTC and might favor a particular orientation of A2602 or of the L27 N‐terminus, key residues that modulate the accessibility of the PTC for aminoacyl‐tRNA or release factor binding (Baram & Yonath, 2005), thereby attenuating translation via a two‐way crosstalk.

Our results show that a β‐barrel protein starts to fold as α‐helix inside the ribosomal exit tunnel. Because α‐helices form through local interactions, their cotranslational folding is likely facilitated by the vectorial emergence of the nascent chain, compaction of the nascent chain, and flexible interactions with ribosomal residues in the exit tunnel. This finding provides a simple rationale for the existence of the potential dormant α‐helical domains in globular proteins regardless of their final fold (Chen *et al*, 2010), as originally hypothesized by Lim (Lim, 1978; Lim & Spirin, 1986). While refolding studies provided no evidence for α‐helical folding intermediates in β‐sheet proteins in solution, our data show that α‐helices may readily form in the exit tunnel of the ribosome. Formation of a non‐native α‐helix may protect the emerging N‐terminal residues from misfolding and entropically facilitate the search of native interactions as the nascent chain gradually moves into the vestibule, which offers enough space for the β‐hairpin to fold. The β‐hairpin is likely to form a nucleation core to guide folding once the peptide is released, thereby preventing misfolding on the path to the native state. Because folding of CspA is intrinsically very rapid, packing of the emerging β‐strands may occur sequentially while the peptide moves out of the tunnel, akin to sequential packing of α‐helical elements in HemK (Liutkute *et al*, 2020a). As the nascent chain remains highly dynamic, the energetic barriers between different conformations are low, which may help to avoid misfolding and aggregation.

## Materials and Methods

### Cloning, expression and purification of CspA

The wild‐type CspA (UniProtKB ID: P0A9X9; PDB ID: 1mjc) was derived from *E. coli* (strain K12) and cloned into pet24a(+) vector with a kanamycin (Kan) resistance cassette. CspA variants (Appendix Table S5) were produced by site‐directed mutagenesis protocol QuickChange™ generating CspA W11L and W11L/F20W variants (Appendix Table S5). To express CspA, a C‐terminal GST‐tag was added followed by a His‐tag with six His residues (Appendix Table S5). Overexpression was performed in *E. coli* BL21(DE3) in LB media with 50 μg/ml Kan, based on a previously published protocol (Chatterjee *et al*, 1993). CspA expression was induced with 1 mM IPTG at cell density OD_600_ ~0.5, and cell harvested after 2 h at 37°C by pelleting at 4,000 *g*, rotor JLA 8.1 (Beckman Coulter) for 15 min. Pellets were flash frozen and stored at −80°C.

Cell pellets were resuspended in buffer A (50‐mM HEPES pH 7.5, 70 of mM NH_4_Cl, 30 mM of KCl, 7 mM of MgCl_2_, 10% glycerol, 5 mM of β‐mercaptoethanol; 2 ml per 1 g of cells), complemented with Dnase I (Jane Bioscience) (40 units per 10 g of cells) and Protease Inhibitor Cocktail cOmplete (Roche Diagnostics GmbH) (1 tablet per 50 ml of buffer). Cells were homogenized using a glass rod homogenizer and lysed using an EmulsiFlex‐C3 (Avestin Europe GmbH) according to the manufactures protocol. Cell lysate was cleared by centrifugation at 50,000 *g* using a JA25.50 rotor (Beckman Coulter) for 30 min at 4°C. The lysate was loaded onto Protino Ni‐IDA matrix (2 g; Macherey Nagel) pre‐equilibrated with the above buffer. Protein was eluted with the same buffer containing 200 mM imidazole and dialyzed in D‐tube Mega dialyzers (MWCO3.5 kDa; Novagen) over 16 h into the tag cleavage buffer (50 mM Tris‐HCl pH 8.0, 150 mM of KCl, 10% glycerol, 0.5 mM of EDTA, 0.5 mM of DTT) in the presence of TEV protease (Sigma Aldrich) according to manufacturer’s instructions. The cleaved His‐tag and TEV protease were removed by purification over Ni‐IDA Protino matrix (Macherey Nagel) using buffer A for column equilibration. The dialyzed protein sample was loaded onto the column and the flow‐through containing the untagged CspA was collected for further purification through anion exchange chromatography, using a HiTrap Q column (1 ml; GE Healthcare Life Sciences) on an Äkta FPLC system (GE Healthcare Life Sciences) equilibrated with 25 mM HEPES pH 7.5 and 10% glycerol. At 15% of elution buffer containing 25 mM HEPES pH 7.5, 10% glycerol and 1 M KCl, CspA protein does not bind to the column and can be collected in the flow‐through during loading step, while the contaminants remain bound to the column. Collected CspA protein was exchanged into 50 mM HEPES pH 7.5, 70 mM of NH_4_Cl, 30 mM of KCl, 7 mM of MgCl_2_, 10% glycerol during the concentration step with Vivaspin MWCO 3.5 kDa (Sartorius AG). All protein purification steps were monitored by SDS‐PAGE and absorbance measurements at A280 using Nanodrop (Thermo Fisher Scientific).

### Circular dichroism spectroscopy and thermal denaturation experiments

Circular dichroism (CD) thermal unfolding transitions were recorded with a Chirascan model dichrometer (Applied Photophysics, Leatherhead, UK). Thermal denaturation experiments were collected in single‐wavelength mode (222 nm) from 20°C to 85°C in 2°C increments using a quartz cuvette of 1 mm path length and protein solutions at a concentration of 20 µM. The temperature was measured directly in the cuvette using a Peltier thermoelement. At each temperature increment, the machine unit ellipticity Θ was averaged for 30 s after an incubation time of 60 s at the target temperature and corrected for background (buffer without protein). Thermal unfolding transitions were measured in CD buffer (150 mM NaH_2_PO_4_, 30 mM NaF, pH 7.5) as well as in buffer TAKM_7_ (50 mM Tris–HCl, 70 mM of NH_4_Cl, 30 mM of KCl, 7 mM of MgCl_2_, pH 7.5) to compare the protein stability in the conditions used in translation experiments.

### Components of translation assay

*Escherichia coli* 70S ribosomes, fluorescence labeled (with Bodipy FL (BOF) or ATTO655) Met‐tRNA^fMet^, initiation and elongation factors, and aa‐tRNA used in *in vitro* translation reactions were prepared in‐house as described (Rodnina & Wintermeyer, 1995; Milon *et al*, 2007; Doerfel *et al*, 2013; Mittelstaet *et al*, 2013; Holtkamp *et al*, 2015). To form the initiation complexes (IC), 70S ribosomes (1 µM), mRNA (4 µM), BOF‐[^3^H]Met‐tRNA^fMet^ (or ATTO655‐[^3^H]Met‐tRNA^fMet^ for PET‐FCS experiments) (1.5 µM), and initiation factor mix (IF1, IF2, and IF3—2.25 μM each) were incubated in TAKM_7_ (50 mM Tris–HCl pH 7.5, 70 mM of NH_4_Cl, 30 mM of KCl, 7 mM of MgCl_2_) with 2 mM of DTT and 2 mM of GTP at 37°C for 45 min. The factor mix was formed by combining phosphoenolpyruvate (PEP; 6 mM), pyruvate kinase (PK; 2% (v/v)), EF‐Tu‐GDP (116 µM), and EF‐G (4 µM) and incubating in the same buffer at 37°C for 15 min. To form ternary complexes (TCs), aminoacyl‐tRNA (200 µM) was added to the factor mix and incubating for 1 min at 37°C.

CspA mRNAs were prepared by *in vitro* transcription as described (Agirrezabala *et al*, 2017; Liutkute *et al*, 2020a). The DNA templates for transcription were generated using PCR with a commercially available T7 forward primer (Eurofins Genomics, Ebersberg) and unique reverse primers for different construct lengths coding for the full‐length 70 aa, as well as shortened 27 aa, 19 aa, and 14 aa‐long peptides (Appendix Table S6). After purification with RNeasy kit (Qiagen) according to the manufacturer’s protocol, the mRNA quality was controlled by PAGE under denaturing conditions (8 M urea), which showed > 90% mRNA purity.

### *In vitro* translation kinetics

The *in vitro* translation reactions contained IC (final concentrations of 0.08 μM), TC (49 μM calculated using EF‐Tu concentration) and EF‐G (0.84 μM) in HiFi buffer (50 mM Tris–HCl pH 7.5, 70 mM of NH_4_Cl, 30 mM of KCl, 3.5 mM of MgCl_2_, 8 mM of putrescine, 0.5 mM of spermidine, 1 mM of DTT, 1 mM of GTP). Translation reactions were started by quickly mixing IC and TC mixtures that were prewarmed for 1 min at 37°C and then incubating for 1 s to 40 s. Translation was stopped by flash‐freezing samples in liquid nitrogen. The peptidyl*‐*tRNA was hydrolyzed by incubation with 0.33 M NaOH at 37°C for 30 min, followed by pH neutralization with addition of HEPES to a final concentration of 0.31 M. After adding gel loading buffer (24% (w/v) glycerol, 2% (v/v) β‐mercaptoethanol, 0.1 M Tris–HCl pH 6.8 in 10% SDS) to final concentration of 50% v/v the samples were boiled at 70°C for 10 min and stored at −20°C until further analysis.

The peptides synthesized by *in vitro* translation were analyzed using three‐layered Tris‐Tricine‐SDS PAGE consisting of the 16.5% separating (49.5% T, 6% C), 10% spacer, and 4% stacking gels (Schagger & von Jagow, 1987; Schagger, 2006). The N‐terminal Bodipy FL or ATTO655 dyes were used to detect translation products at 488 nm or 680 nm wavelength, respectively, using a Fujifilm FLA‐9000 fluorescence scanner. The individual translation bands were quantified with Fuji7000 scanning software. For FPA, the fraction of the full‐length product was calculated by dividing the full‐length band intensity by the sum of the full‐length and arrested band intensities. Gel quantifications were plotted and fitted using GraphPad Prism version 8.3 software. Average translation times were calculated by fitting time courses with a delay‐exponential model (GraphPad Prism), and the average rate of translation in amino acids/s was calculates for each construct by dividing the nascent chain length by the translation time. The simulated traces for CspA11 and CspA14 formation were produced in GraphPad Prism v8.3 using the average translation time determined for CspA19.

### Following translation by stopped‐flow

An SX20 stopped‐flow fluorimeter (Applied Photophysics, Leatherhead, UK) in single‐step mixing mode was used to monitor the *in vitro* translations in real‐time at 37°C. The N‐terminal BOF was excited at 465 nm and the emission monitored after passing a KV500 cut‐off filter (Schott). After mixing IC and TC, fluorescence was recorded for up to 35 s. Values < 1 ms were excluded from analysis. In each case, 5–6 technical replicates were acquired per biological replicate and at least 5 technical replicates and 3 biological replicates were averaged to obtain the final traces. PET efficiencies were calculated by normalizing W11 or F20W traces by the W11L control for each chain length. The change in PET after the release of CspA70 from the ribosome was monitored after rapidly mixing the complexes with ArfB (1 µM). GraphPad Prism version 8.3 was used for plotting and analyzing the data with in‐built exponential equations, where possible (Appendix Table S2). Time courses of CspA19 PET efficiency were fit with Table Curve 2D version 5.01 software (SYSTAT software Inc.) using a user‐defined function:

(1) $$\begin{array}{c} F1=IF\left(X\leq a,b‐Y,b+c_{1}‐c_{1}*e^{\left(‐k_{1}*\left(X‐a\right)\right)}+c_{2}‐c_{2}*e^{\left(‐k_{2}*\left(X‐a\right)\right)}\right)‐Y)\\ Y=IMPLICIT\left(F1,O,10,1e^{‐8}\right) \end{array}$$

where a is the delay time, *b* is the plateau, *c*_1_ is first exponent amplitude, *k*_1_ is the apparent rate constant of the first exponent, *c*_2_ is the second exponent amplitude, and *k*_2_ is the associated apparent rate constant.

### FPA measurements

The CspA FPA constructs contained the CspA sequence (1–70 aa) at the N‐terminus, followed by a 17 amino acid SecM stalling peptide sequence (71–87 aa) (Nakatogawa & Ito, 2002) and a 23 aa LepB sequence at the C‐terminus (Nilsson *et al*, 2015) (Appendix Table S7). The full‐length plasmid was synthesized by Eurofins Genomics (Ebersberg, Germany) in a background of pEX‐A128 vector with a cassette for ampicillin resistance. Truncations from the C‐terminus of CspA sequence were performed in 2 aa steps from 70 to 3 aa using primer sequences listed in Appendix Table S8. All cloned variants were verified with Sanger sequencing (Microsynth AG, Göttingen, Germany). DNA templates for mRNA transcription were amplified using commercially available standard T7 forward primer and LepB specific reverse primer (5′‐ATGGATGCCGCCAATGCGAC) (Eurofins Genomics, Ebersberg).

### RNC preparation

For the FCS experiments, the ribosome nascent chain complexes (RNCs) were generated with ATTO655‐[^3^H]Met‐tRNA^fMet^ as the initiator tRNA and purified from the translation mixture by sucrose cushion centrifugation, using a 40% (w/v) sucrose in HiFi buffer. The samples were centrifuged at 200,000 *g* in TLA‐100 rotor (Beckman Coulter, USA) at 4°C for 40 min. RNC pellets were dissolved in HiFi buffer and concentration was assessed by scintillation counting using ^3^H‐label QuantaSmart (Perkin Elmer). Purification quality control was carried out by SDS‐Tricine PAGE. RNCs were stored at −80°C.

For cryo‐EM, the RNCs were purified via size exclusion chromatography. CspA27 and CspA70 were translated as described above for 5 min at 37°C. The Biosuite 450HR size exclusion column (Merck KGaA, Darmstadt, Germany) with a pore size of 8 µm was used to separate RNCs from translation components in HiFi. Fractions containing RNCs were collected and stored at −80°C until further use. RNC quality control was performed by scintillation counting with QuantaSmart (Perkin Elmer).

### PET‐FCS

For FCS measurements, the RNCs were diluted to 5–7 nM in HiFi buffer. Measurements were performed using a confocal microscope FCS set‐up (Micro Time 200, Olympus IX73) (PicoQuant, Berlin, Germany) at 636.5 nm wavelength continuous wave laser with power ~40 μW and 1–1.5 molecules per observation volume (Liutkute *et al*, 2020a). Data processing and autocorrelation function (ACF) calculations were done using SymPhoTime 64 software (PicoQuant, Berlin, Germany). Each sample was subject to 4 measurements of 10 min, which were averaged. RNCs of each CspA variant were independently prepared and measured 2 times, generating an overall of 8 technical replicates for each final ACF.

Fitting of initial ACFs was carried out using a model (Liutkute *et al*, 2020a) for single species diffusion with two relaxation rate constants, a triplet rate constant, and a diffusion rate constant,

(2) $$G\left(\tau \right)=\left(1+c_{1}e^{‐k_{1}t}+c_{2}e^{‐k_{2}t}\right)\left(\frac{1‐F+Fe^{‐k_{f}t}}{1‐F}\right)\left(\frac{1}{N}\right){\left(1+k_{d}t\right)}^{‐1}$$

where *k*_1_ and *k*_2_ are apparent relaxation rate constants with respective amplitudes *c*_1_ and *c*_2_, *N* is the average number of molecules in the confocal volume, *F* is the amplitude for the triplet component with rate constant *k*_f_, and *k*_d_ is the inverse diffusion time. All curves were fitted independently of each other and results are displayed in Appendix Table S4.

### Cryo‐EM and image processing

Sample aliquots (4 μl) were applied to glow‐discharged Quantifoil R 2/1 holey carbon grids coated with a thin layer of carbon prepared in‐house. After incubation for 20 s and blotting for 2 s, the grids vitrified in a FEI Vitrobot (ThermoFisher) were transferred to a Titan Krios (ThermoFisher) electron microscope operated at 300 kV. Images were acquired using a Falcon III detector at a magnification of 130.841, yielding a pixel size of 1.07 Å. Data was collected using the automated data collection software EPU (Thermo Fisher). Multiframe movies (40 frames, dose per frame: 2.2 e‐/Å2) were recorded with the detector in integrating mode, using a nominal defocus (dF) range of −0.5 to −2.2 μm. The movie frames were aligned (4 × 4 patches) and dose weighted using MotionCor2, version 1.0.5 (Zheng *et al*, 2017). Contrast transfer function (CTF) parameters were estimated using the non‐dose‐weighted images by Gctf, version 1.06 (ref. (Zhang, 2016), and the ribosomal particles were automatically picked from the aligned micrographs by Gautomatch, version 0.53 (https://www2.mrc‐lmb.cam.ac.uk/research/locally‐developed‐software/zhang‐software/). All further image processing was carried out using the Relion software package, version 3.07 (Zivanov *et al*, 2018).

For CspA27, after initial visual inspection for good ice quality and CTF, as well as absence of astigmatism or significant drift, the selected 14,303 movie stacks yielded an initial data set of 1,860,401 particles. This set, decimated by a factor of two, was subjected to an initial round of reference‐free 2D classification. The selected 1,404,270 particles gave rise to a refined 4.30 Å 3D reconstruction after auto‐refinement. These particles were subsequently used for treatment of structural heterogeneity as follows (Appendix Fig S3): First, the initial dataset was subjected to 3D classification without the use of any mask: the states thus isolated represented classical state‐like ribosomes with A/A and P/P tRNAs (3%), hybrid state‐like ribosomes with A/P and P/E tRNAs (6.6%) and a state with a P‐site tRNA only (81%). The two additional subsets (3.7% and 5.5%) largely showed density corresponding to the 50S subunit.

At this point, an additional round of 2D classification was performed on the most populated subclass to filter out misaligned images or any remaining false positives. After particle sorting and manual curation of ribosome particles belonging to good classes, 1,092,742 original sized particles were re‐extracted, and the images refined and subjected to Bayesian particle polishing (for a final accumulative electron dose of ~55 e^−^/Å^2^) and CTF refinement (to estimate per‐particle dF values). The approach produced a map with an estimated resolution of 2.83 Å at an FSC cutoff of 0.143.

To identify the subset of ribosomes containing tRNA^Ser^, which is the tRNA which should be in the P site of CspA27 complex, we then performed a round of focused 3D classification with the previously refined angular parameters. We used a soft‐edged mask covering the mRNA codon at the P‐site, as well as density that accounted for the variable arm of tRNA^Ser^, which is a large stem–loop structure consisting of several nucleotides between the anticodon and the TψC stem. This strategy provides a straightforward way to take advantage of the fact that in *E. coli*, this structure is only present in tRNA^Ser^, tRNA^Leu^ and tRNA^Tyr^, which in in the CspA sequence appear at codons 2, 27, 35, 44, 52, 57, and 69 (tRNA^Ser^), 45 and 70 (tRNA^Leu^), and 42 (tRNA^Tyr^). This procedure yielded a 31.7% class of 348,868 particles with strong density for the variable loop of the tRNA, which was refined to yield a 2.74 Å map after another round of CTF refinement. This second CTF refinement included the use of the image shifts applied while acquiring data to group images for beam tilt estimation, as well as per‐particle astigmatism estimation.

Next, hierarchical focused 3D classifications were performed on the selected subset in order to improve the density corresponding to the nascent chain. The masks used encompassed not only the tRNA but also the ribosomal exit tunnel. In addition, partial signal subtraction was applied to minimize reference/experimental projection comparison inconsistency. Initial sorting into five groups yielded a class (27.1%) that showed continuous density for the nascent chain along with further improved signal‐to‐noise. While the rest of the subsets were ignored, the subtracted ribosome density for this class was restored and the selected particles refined to 2.85 Å in order to proceed with the second round of masked classification with signal subtraction. As previously, all orientations were kept fixed at the values determined in the refinement of the consensus reconstruction. The final round of 3D refinements for the three classes that were thus generated (comprised by 35,573, 44,182, and 15,459 particles, respectively) yielded maps at ~3 Å, 3.05 Å and 3.2 Å resolution by FSC at 0.143 after subtracted signal was reverted (Appendix Fig S4). Angular accuracies of 0.275°, 0.283°, and 0.314° were achieved in the final auto‐refinements for CspA‐27 class1, class2, and class3, respectively.

For CspA70, following quality control inspection, 955,073 particles from 6,798 movie stacks were subjected to reference‐free 2D classification. The 814,221 selected particles gave rise to a refined 2.87 Å 3D reconstruction and were used for treatment of structural heterogeneity as follows (Appendix Fig S8). As with CspA27, the initial dataset was subjected to 3D classification without the use of any mask. The captured states represented classical state ribosomes with A/A and P/P tRNAs (10.3%), hybrid state‐like ribosomes with a P/E tRNA (4.8%) and two substates with a P‐site tRNA only (81% and 0.6%). 682,602 particles from the major subclass were then refined and subjected to Bayesian particle polishing (for a final accumulative electron dose of ~55 e^−^/Å^2^) and CTF refinement (to estimate per‐particle dF values and astigmatism, as well as to estimate the beam tilt over the entire data set) to produce a 2.24 Å resolution map.

As in the case of CspA27, the classification workflow for CspA70 then intended to isolate the subset of ribosomes containing the in‐register tRNA (tRNA^Leu^ in this case, which also presents a variable loop). Next, the workflow process was designed to identify complexes that showed sufficient signal for the full‐length nascent chain, continuous for the entire segment from C‐term to N‐term. As previously, this was achieved in additional multiple runs with different combinations for number of classes, regularization parameter T (tau_fudge) values and/or masks that accounted for the tunnel in a hierarchical manner.

To isolate the subset of tRNA^Leu^ containing complexes, two consecutive rounds of focused 3D classifications were performed first (the second round involving signal subtraction) using a soft‐edged mRNA‐tRNA mask that also accounted for the variable arm present in tRNA^Leu^. 159,979 particles were isolated and refined to yield a 2.53 Å resolution map. Hierarchical signal‐subtracted classifications then performed on this subset made use of a mask encompassing the anticodon stem loop (ASL) of the tRNA, as well as the ribosomal tunnel and exit port. The major class isolated in the initial sorting (30.9%) was refined to 2.78 Å after signal restoration; it showed continuous density for the nascent chain, but was subjected to the final round of masked classification as residual heterogeneity at the PTC level (mostly scattered signal for L27) was detected by visual inspection. 3D refinements for the two subsets thus generated (comprised by 23,782 and 26,765 particles) yielded maps at a resolution of ~2.9 Å (Appendix Fig S9). Angular accuracies of 0.27° and 0.267° were achieved in the auto‐refinements for CspA70‐1 and −2 reconstructions, respectively.

Note that multiple additional runs (not shown in Appendix Figs S3 and S8) with different numbers of classes, regularization parameter T (tau_fudge) values, and/or masks were performed at different stages of the sorting procedure in both data sets. During these runs, orientation searches were performed exhaustively, that is, every 0.9 degrees (restricted to local searches) or kept fixed at the orientations from the consensus refinements. Separate 3D auto‐refinements were performed for each of the corresponding subsets after each round of classification. The visual inspection of the resulting 3D reconstructions (mostly signal quality for nascent chain as well as other elements lining the tunnel wall and at the PTC), and the presence/absence of redundant or empty classes, in conjunction with local resolution assessment helped in planning the best strategy to deal with the structural heterogeneity. Local resolution variability (Appendix Figs S4 and S9) was estimated via ResMap, version 1.1.4 (Kucukelbir *et al*, 2014) and visualized by coloring the corresponding maps in Chimera, version 1.13.1 (Pettersen *et al*, 2004).

### Model building and refinement

The atomic models were generated using the *E. coli* 70S ribosome structure PDB‐6ore (Fu *et al*, 2019) as initial framework. Rigid body fitting of the models was carried out using Chimera, and the rest of the structure, including missing parts of tRNAs (i.e., variable loops) and nascent chains built using Coot, version 0.9 (Emsley & Cowtan, 2004). In CspA70 reconstructions, the N‐terminal fragment aa 5–25 from PDB‐1mjc (Schindelin *et al*, 1994) was rigid‐body docked and used as initial threading template for further building toward the C‐termini via Coot. The model for CspA70‐1 nascent chain does not include the N‐terminal fMet‐Ser‐Gly‐Lys (residues 1–4). Also, CspA70‐2 has fragmented and weak density for the peptide throughout the upper and central regions of the tunnel and was not modeled. The models were improved by iterative cycles of manual rebuilding and refinement into maps sharpened by the Phenix auto‐sharpen tool (Terwilliger *et al*, 2018). The real space procedure in Phenix (Afonine *et al*, 2018b) was used to refine the models and to improve stereochemistry. The restrains used during refinements included the geometric restrain for the ester bond between the 3’O of A (the CCA end of tRNA) and the carbonyl C of the attached Ser (CspA27) and Leu (CspA70). Models were validated throughout refinement and quality of fit assessed using the tools for analysis and validation integrated in the Phenix suite, version 1.18 (Afonine *et al*, 2018a) (Appendix Table S3). The software LocScale for model‐based local density sharpening (Jakobi *et al*, 2017) was used as implemented in the CCP‐EM suite, version 1.3.0 (Burnley *et al*, 2017) to rescale the maps for visualization and figure preparation with Chimera.

### Statistical analysis

Translation kinetics and fluorescence data were analyzed and fitted using GraphPad Prism v8 (using in‐built functions) and when indicated with Table Curve 2D version 5.01 using custom functions (SYSTAT software Inc.). These software packages are available commercially. See Methods sections above for details on image processing and model building and refinement.

## Author contributions

**Xabier Agirrezabala:** Conceptualization; Data curation; Formal analysis; Funding acquisition; Validation; Investigation; Visualization; Writing ‐ original draft; Writing ‐ review and editing. **Ekaterina Samatova:** Conceptualization; Formal analysis; Supervision; Validation; Investigation; Visualization; Writing ‐ original draft; Writing ‐ review and editing. **Meline Macher:** Formal analysis; Investigation. **Marija Liutkute:** Conceptualization; Formal analysis; Validation; Investigation; Methodology; Writing ‐ review and editing. **Manisankar Maiti:** Formal analysis; Investigation; Methodology; Writing ‐ review and editing. **David Gil‐Carton:** Resources; Software; Methodology. **Jiri Novacek:** Resources; Methodology. **Mikel Valle:** Resources; Funding acquisition; Methodology. **Marina V Rodnina:** Conceptualization; Formal analysis; Supervision; Funding acquisition; Writing ‐ original draft; Project administration; Writing ‐ review and editing.

In addition to the CRediT author contributions listed above, the contributions in detail are

XA, ES, and MVR conceived the experiments; ES and M. Macher performed biochemical and kinetic experiments; ML analyzed the data; M. Maiti performed PET‐FCS experiments and analyzed the data; M. Macher performed stopped‐flow experiments; XA, DG‐C, and JN conducted cryo‐EM experiments; XA and MV performed and validated cryo‐EM analysis; XA and MVR wrote the manuscript with input from all authors. All authors discussed the data analysis, critically reviewed the manuscript, and approved the final version.

## Disclosure statement and competing interests

The authors declare that they have no conflict of interest.

## Supporting information



Appendix

Movie EV1

Movie EV2

Movie EV3

Movie EV4

Movie EV5

Movie EV6

Movie EV7

Movie EV8

Movie EV9

Movie EV10

Source Data for Appendix

Source Data for Figure 1

Source Data for Figure 3

## Data Availability

All biochemical data are available in the main text or the Supplementary Materials (Appendix). The cryo‐EM maps have been deposited in the Electron Microscopy Data Bank (EMDB), https://www.ebi.ac.uk/pdbe/emdb, under the following accession numbers: EMD‐12636 (CspA27‐1), EMD‐12928 (CspA27‐2), EMD‐12929 (CspA27‐3), EMD‐13055 (CspA70‐1), and EMD‐12930 (CspA70‐2). Atomic coordinates have been deposited in the Protein Data Bank (PDB), http://www.wwpdb.org, under the following accession numbers: 7NWW (CspA27‐1), 7OIF (CspA27‐2), 7OIG (CspA27‐3), 7OT5 (CspA70‐1), and 7OII (CspA70‐2).
