# Supplementary material for: A switch from α‐helical to β‐strand conformation during co‐translational protein folding
Source: EMBO J. 2022 Jan 7;41(4):e109175. doi: 10.15252/embj.2021109175 (PMC8844987; doi:10.15252/embj.2021109175)
Supplement: Supplementary file 1 — Appendix [file EMBJ-41-e109175-s013.docx]

**APPENDIX FILE**

**Table of contents:**

- **Appendix Figures**. Pages 2-13

Figure S1…………………………….……… *p. 2*

Figure S2…………………………….……… *p. 3*

Figure S3…………………………….……… *p. 4*

Figure S4…………………………….……… *p. 5*

Figure S5……………………………….…… *p. 6*

Figure S6……………………………….…… *p. 7*

Figure S7…………………………….……… *p. 8*

Figure S8…………………………….……… *p. 9*

Figure S9………………………………….… *p. 10*

Figure S10…………………………………... *p. 11*

Figure S11…………………………………... *p. 12*

Figure S12…………………………………... *p. 13*

- **Appendix Tables**. Pages 14-19

Table S1……………………………….……. *p. 14*

Table S2……………………………….……. *p. 15*

Table S3……………………………….……. *p. 16*

Table S4……………………………….……. *p. 17*

Table S5……………………………….……. *p. 18*

Table S6……………………………….……. *p. 18*

Table S7……………………………….……. *p. 19*

Table S8……………………………….……. *p. 19*

- **Appendix References**. Page 20

**APPENDIX FIGURES**

**
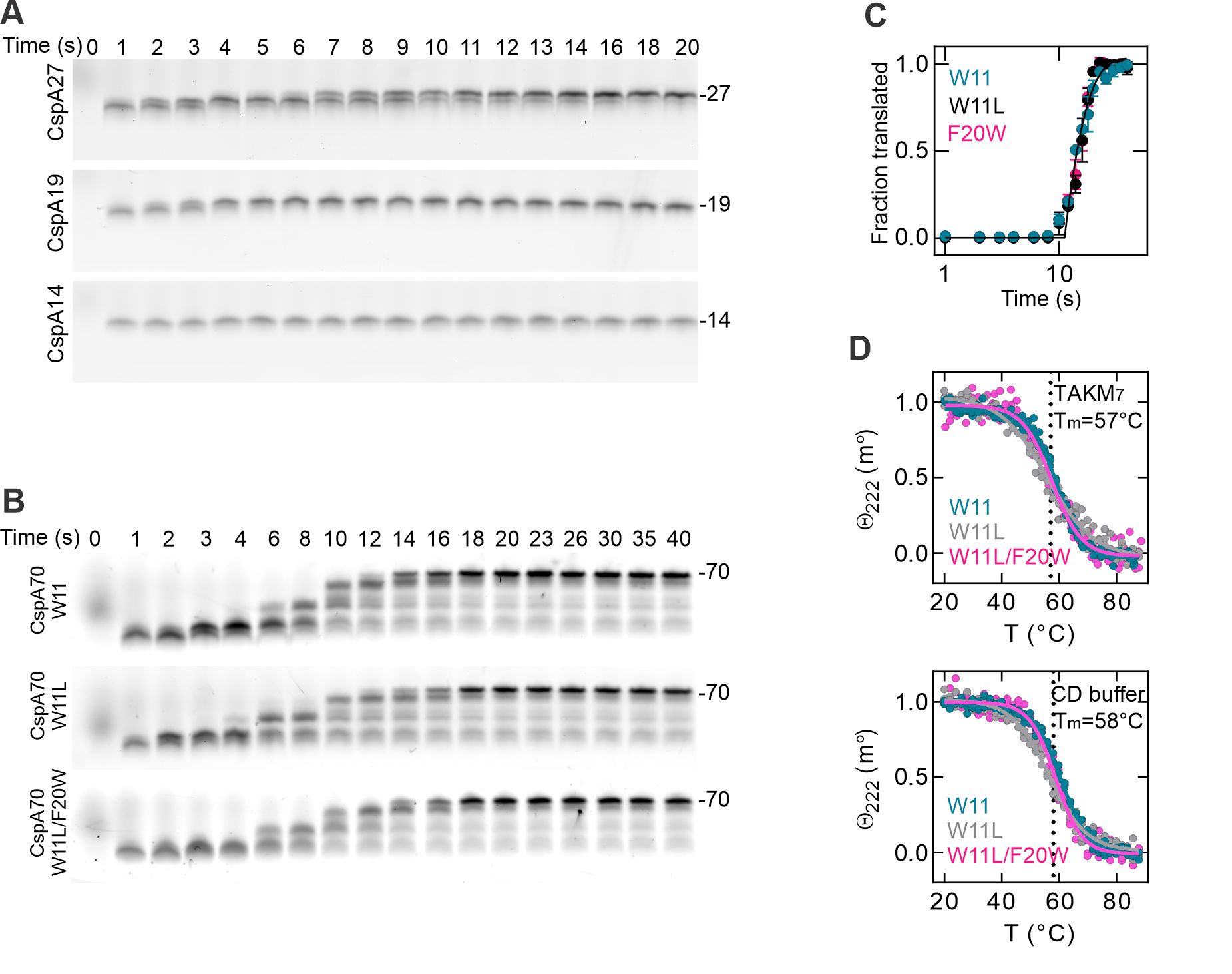
**

**Figure S1.** **Biochemical characterization of CspA.** (A) Translation time courses of CspA constructs of different lengths as indicated. Translation products were separated by SDS-PAGE and visualized by scanning at the excitation wavelength of BOF. Bands below the respective final product show transient accumulation of shorter chains that disappear with time. Translation products <14 aa length do not separate on SDS-PAGE; the respective gel was only used as a guide to assign the transient band appearing upon synthesis of CspA19, e.g., in the middle panel, the upper band corresponds to CspA19, whereas lower band represents a mixture of peptide < 14 aa length. The same two bands are seen at short translation times upon CspA27 synthesis; they disappear with time as 27 aa-length product is synthesized (upper panel). The band density for the final-size product in upper and middle panels was used in Fig 1 to estimate translation rates of CspA27 and CspA19, respectively. Shown are examples of gels; the experiment was repeated 3 times. (B) Time courses of translation of native CspA sequence, as well as W11L and W11L/F20W mutants. The top band was used to determine the time course of synthesis (Fig S2A). The experiments were repeated 3 times. (C) Time courses of translation. Shown are mean values from 3 biological replicates; error bars are SEM (N=3). (D) The thermal stability of wt and mutant CspA variants, determined by measuring the ellipticity at 222 nm with increasing temperature.

**
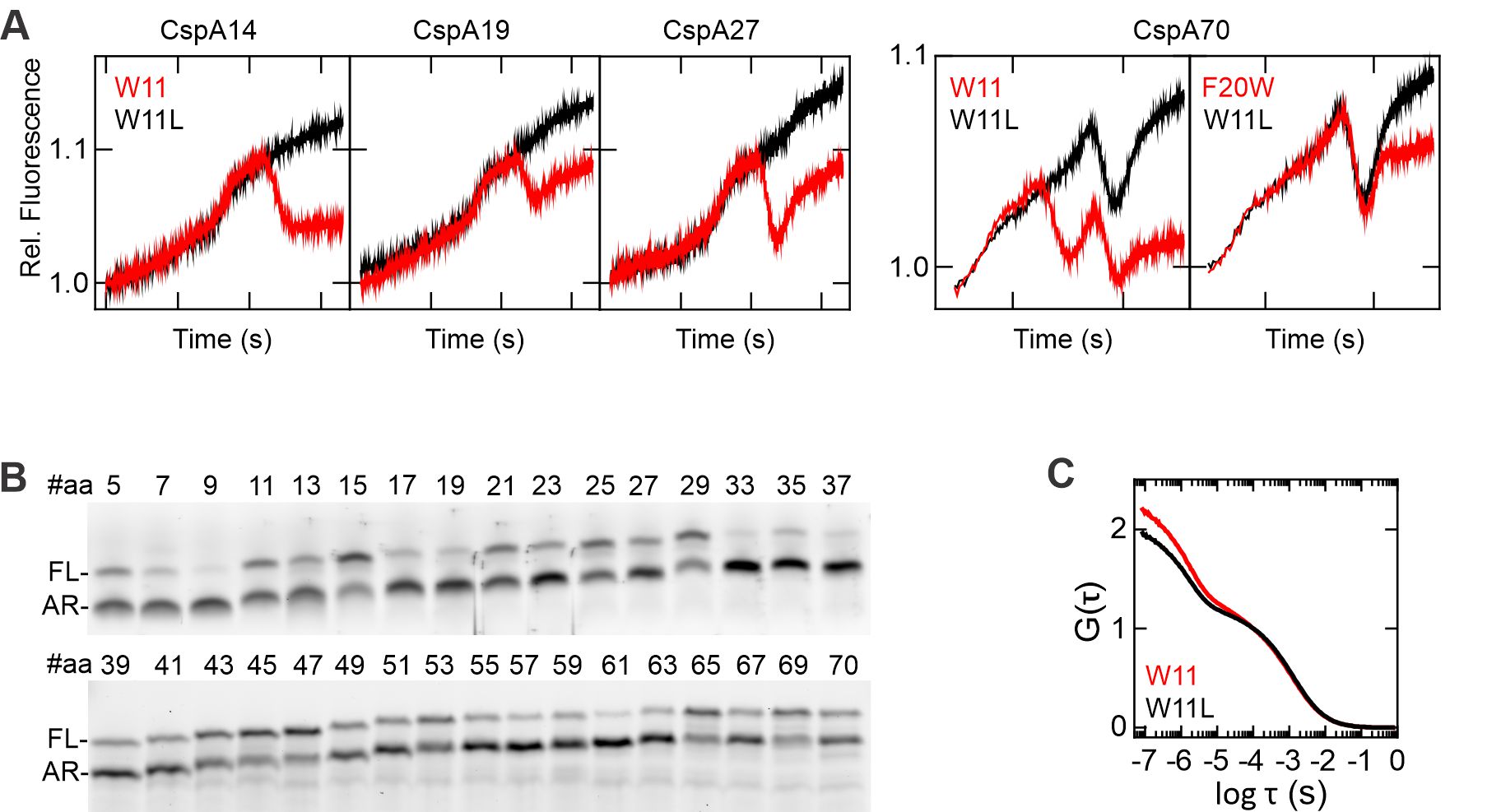
**

**Figure S2. PET and FPA data.** (A) Fluorescence time courses of CspA translation with (red) and without (black) an intramolecular Trp that acts as a quencher of the N-terminal BOF fluorescence. Blue, difference in fluorescence changes with and without Trp. The length of the CspA construct and the position of Trp in the sequence are indicated. (B) FPA analysis. SDS-PAGE of translation products shows two bands which were used to calculate the f_FL_ value in Fig 3. (C) Autocorrelation curves of CspA70 with (red) and without (black) W11. The difference in the amplitudes of the W11 and W11L curves indicates PET of BOF-Met1 by W11. Fitting of the curves to eqn. 1 (Table S4) indicates that the PET change is two-exponential, with the reactions that occur in the µs and sub-µs time scales. Due to two-exponential character of the curves, further analysis towards obtaining the rate constants of the reactions was not feasible.


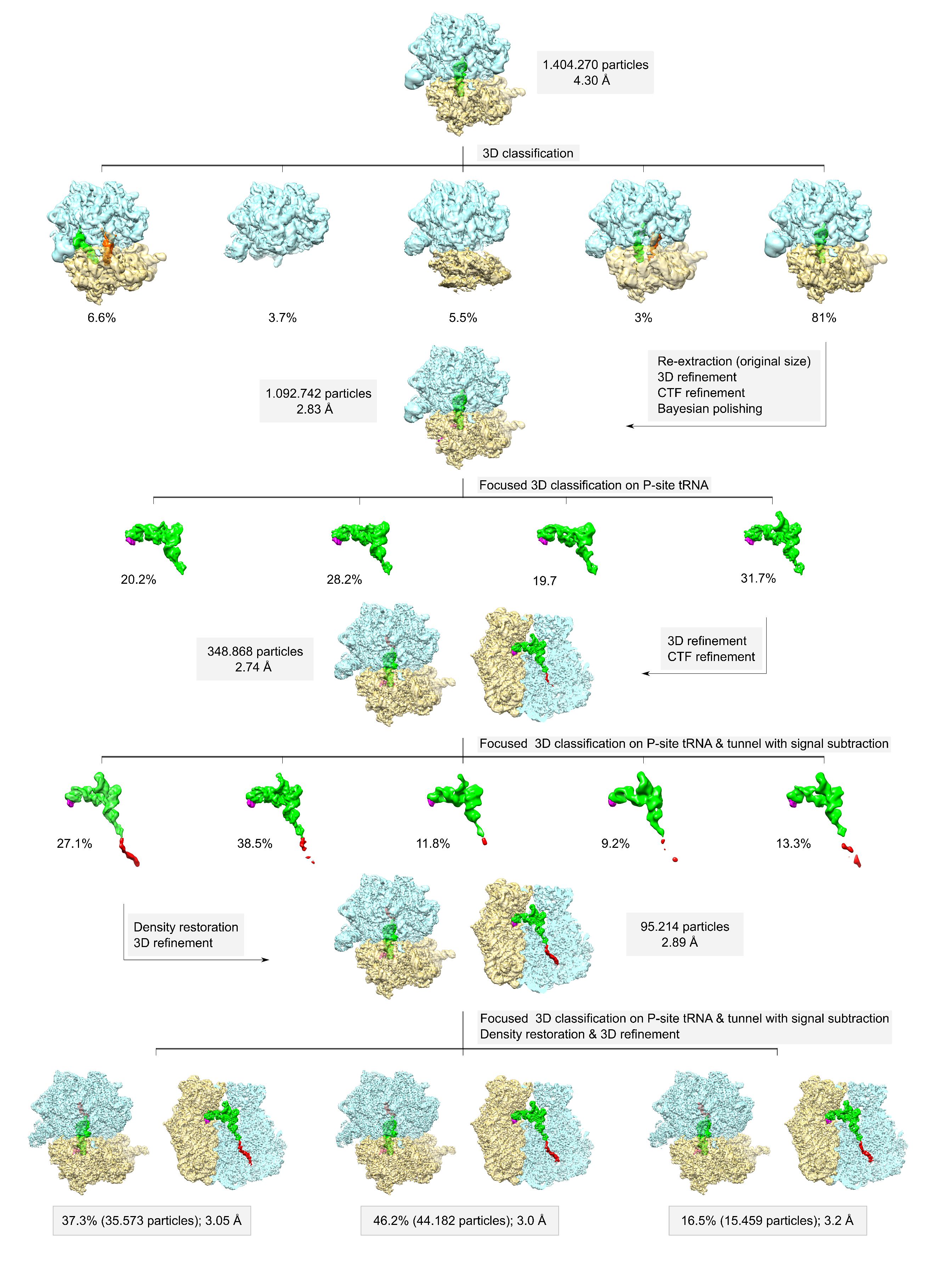


**Figure S3. Overview of particle classification and structure determination for CspA27**. Cryo-EM densities showing 30S (yellow) and 50S (cyan) subunits, A-site tRNA (orange), P-site tRNA (green), nascent peptide (red) and mRNA (magenta). See Methods for details.


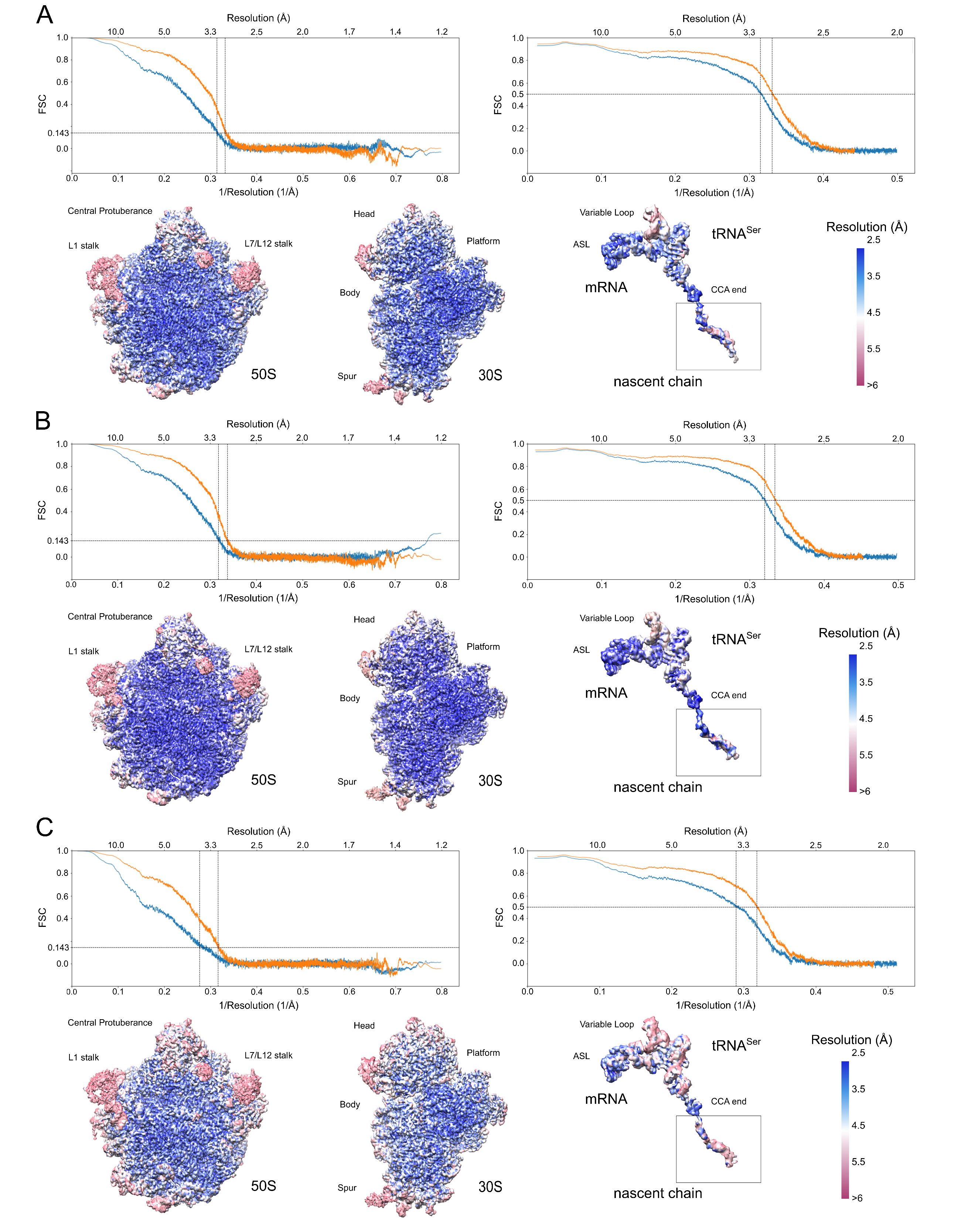


**Figure S4. FSC curves and the local resolution for the reconstructed cryoEM maps.** (A) CspA27-1. (B) CspA27-2. (C) CspA-27-3 The FSC (Fourier Shell Correlation) curve for each of the three subsets is shown as upper panels, the curve for the final model *versus* the original, unsharpened map to the right. The 0.143 and 0.5 cutoffs in the FSC are highlighted. The soft mask used (masked FSC curves, orange) is calculated using the atomic model as previously described (Afonine et al., 2018). The plots were generated by the Phenix evaluation routine mtriage. Lower panels show the local resolution with scale bar providing color scale with resolution in Å.


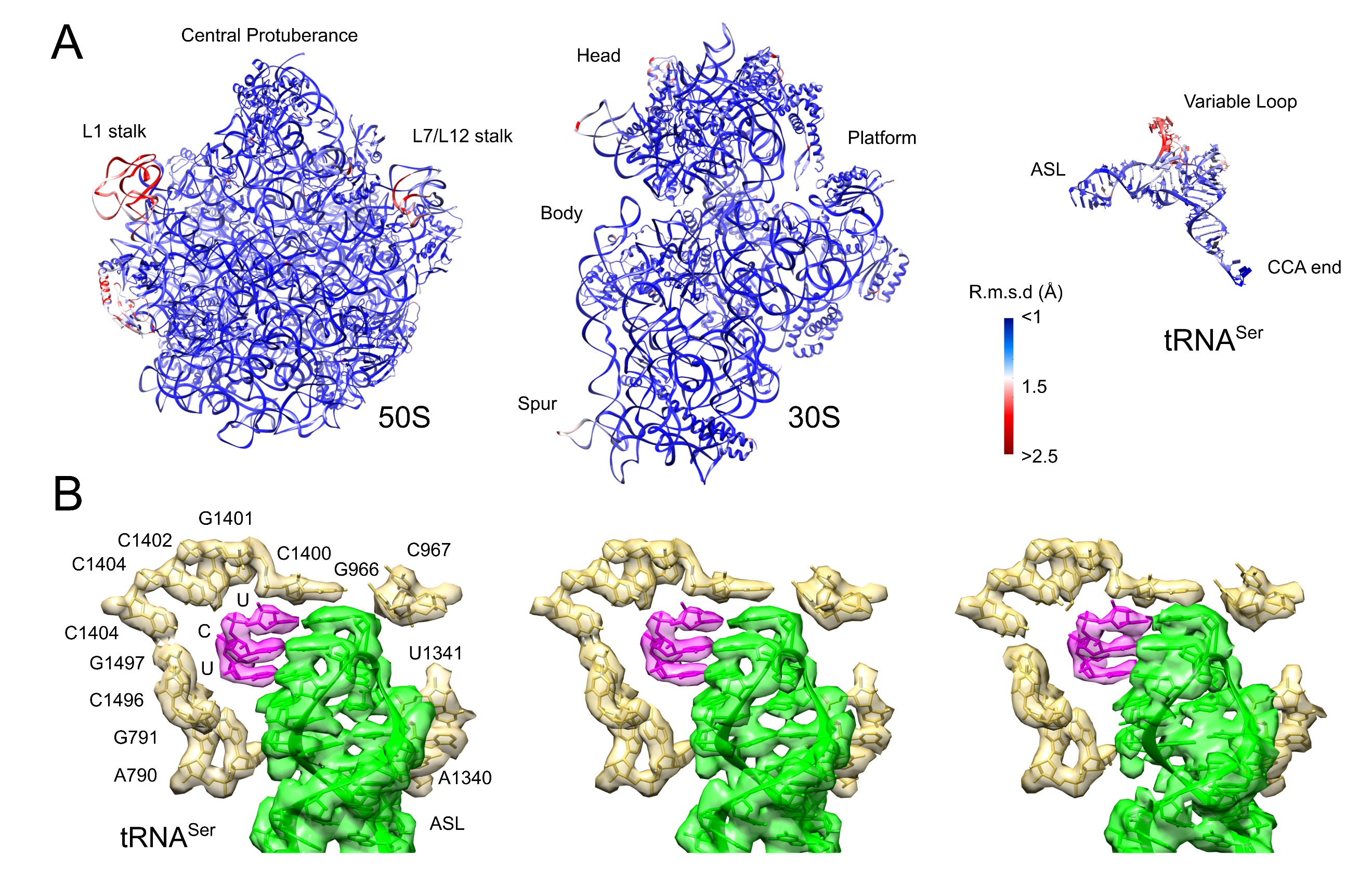


**Figure S5. CspA27 structure comparison.** (A) Superposition of the structures show a root mean square deviation (rmsd) below 1.5 Å for most atoms, emphasizing that most nucleobase and residues have very similar conformation. Changes are limited to inherently flexible domains of the ribosome (such as peripheral parts and both stalks), as well as the variable of loop of the tRNA^Ser^. The data support the notion that structural flexibility is an inherent property of this loop, which likely samples a relatively wide range of conformational states even bound to the ribosome. Scale bar shows color scale with resolution in Å. (B) Close-up view of the codon-anticodon duplexes for CspA27 conformations 1 to 3 (from left to right) showing the framework of interactions at the tRNA anticodon stem-loop region.


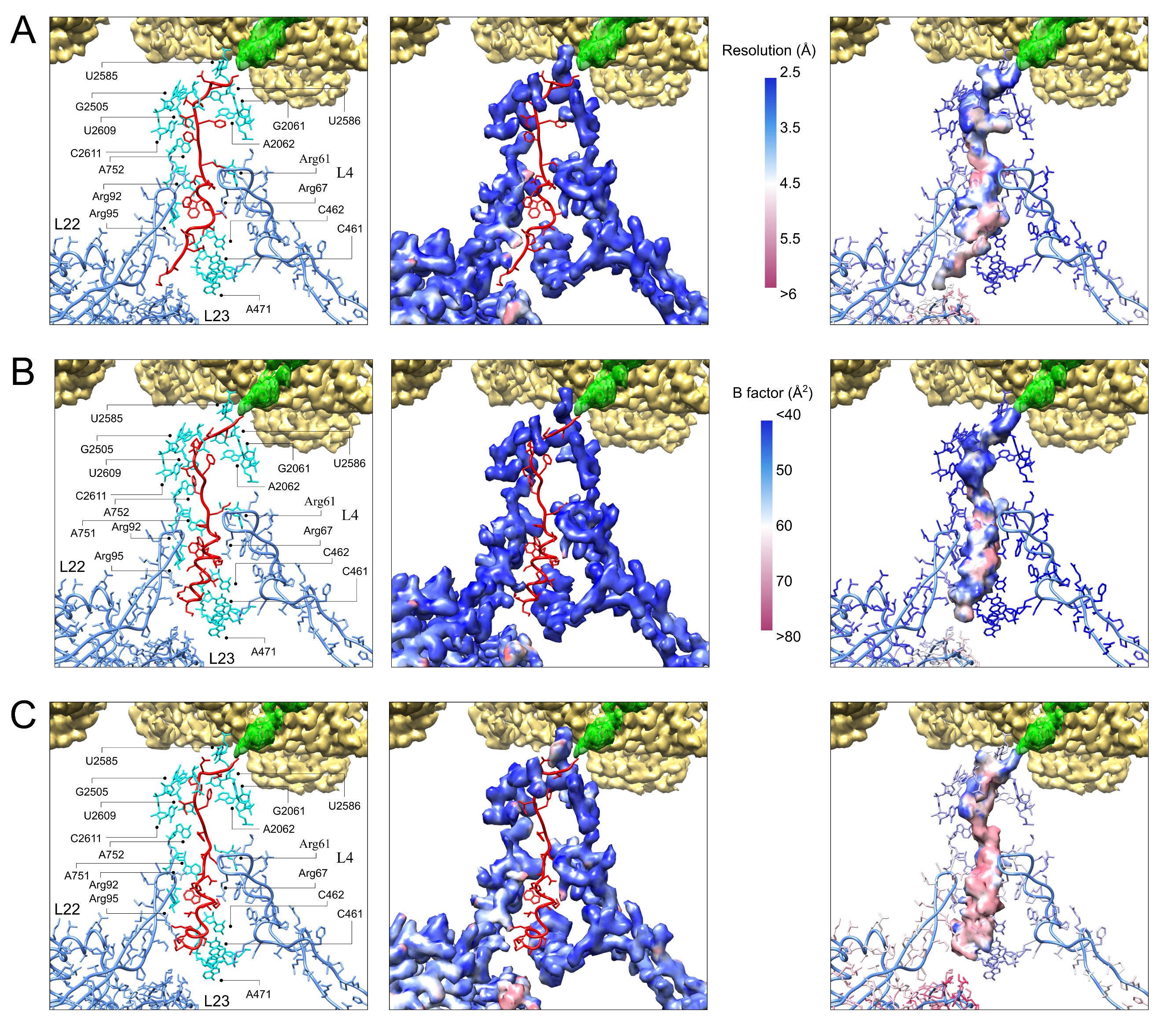


**Figure S6**. **The polypeptide exit tunnel in CspA27 structures, Related to Figure 2**. (A) CspA27-1. (B) CspA27-2. (C) CspA27-3. The local resolution for L4 and L22 is shown (central caption), together with the atomic models generated for each of the conformations (left). The captions to the right show the tunnel wall components L4 and L22, colored by B-factor and the local resolution for each of the nascent chains. Scale bars shows color scale with resolution in Å, and B factor in Å^2^.


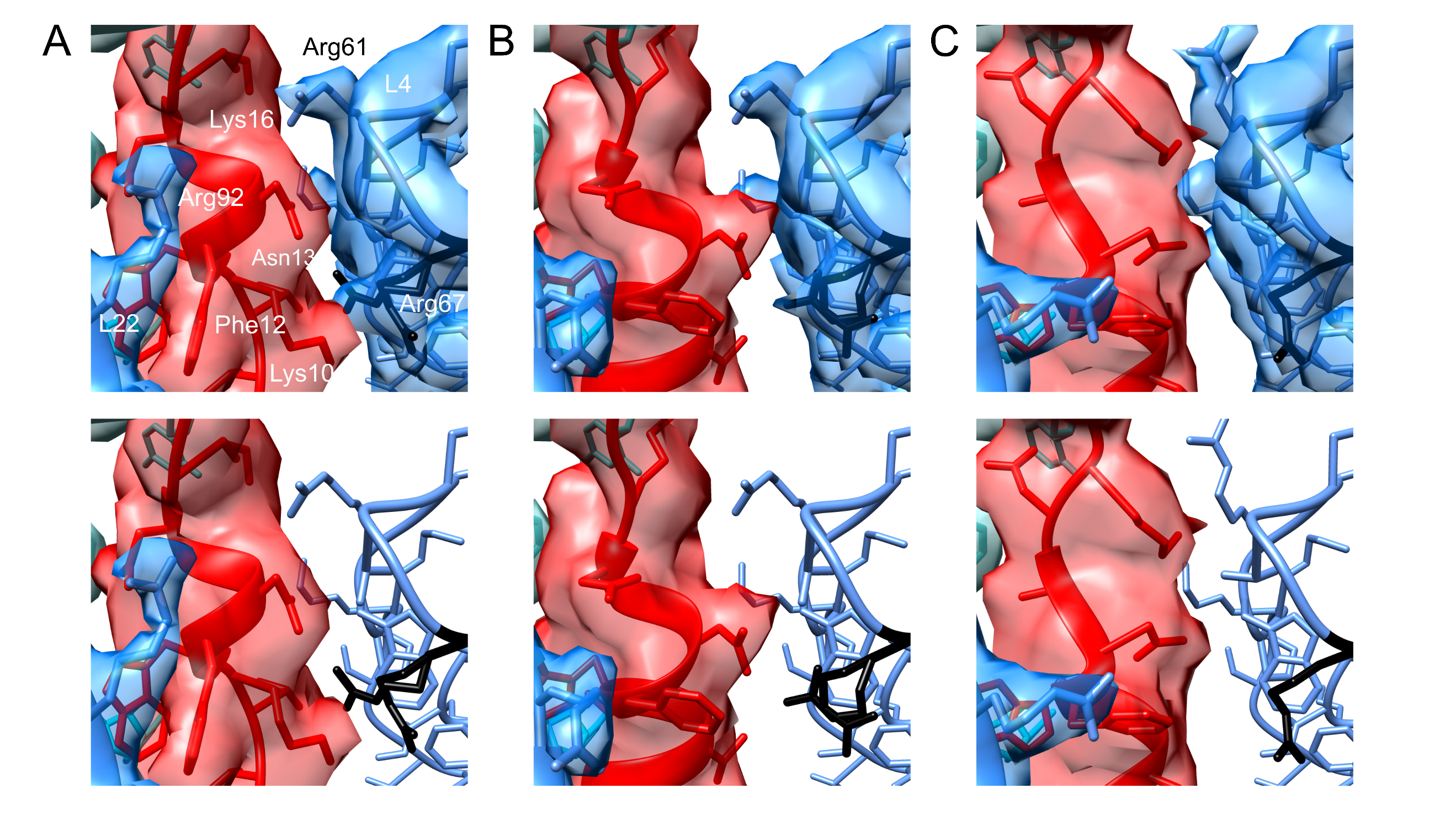


**Figure S7**. **Arg residues in the exit tunnel of CspA27 structures**. (A) CspA27-1. (B) CspA27-2. (C) CspA27-3. Interactions of Arg61 (L4) are described in the main text. Residue Arg67 (L4), highlighted in black, can sample two different conformations in CspA27-1 and CspA27-2, but contacts the nascent chain only in CspA27-1. This conformation is not visited in CspA27-3, where Arg67 only adopts only the most stable, predominant conformation. The density for L4 is omitted in lower panels for clearer visualization of the Arg61 and Arg67 orientation.


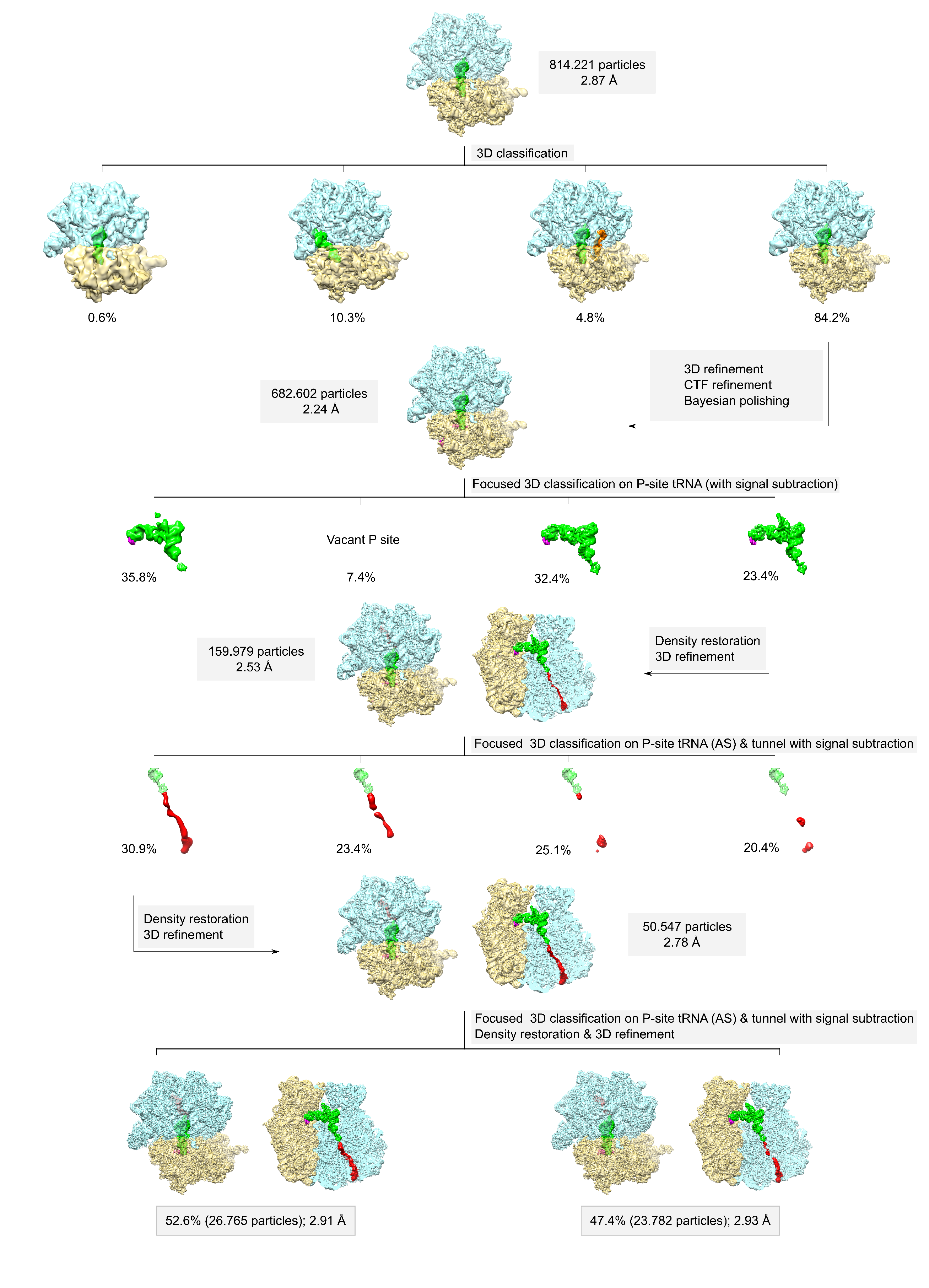


**Figure S8. Overview of particle classification and structure determination for CspA70**. Cryo-EM densities showing 30S (yellow) and 50S (cyan) subunits, A-site tRNA (orange), P-site tRNA (green), nascent peptide (red) and mRNA (magenta). See Methods for details.


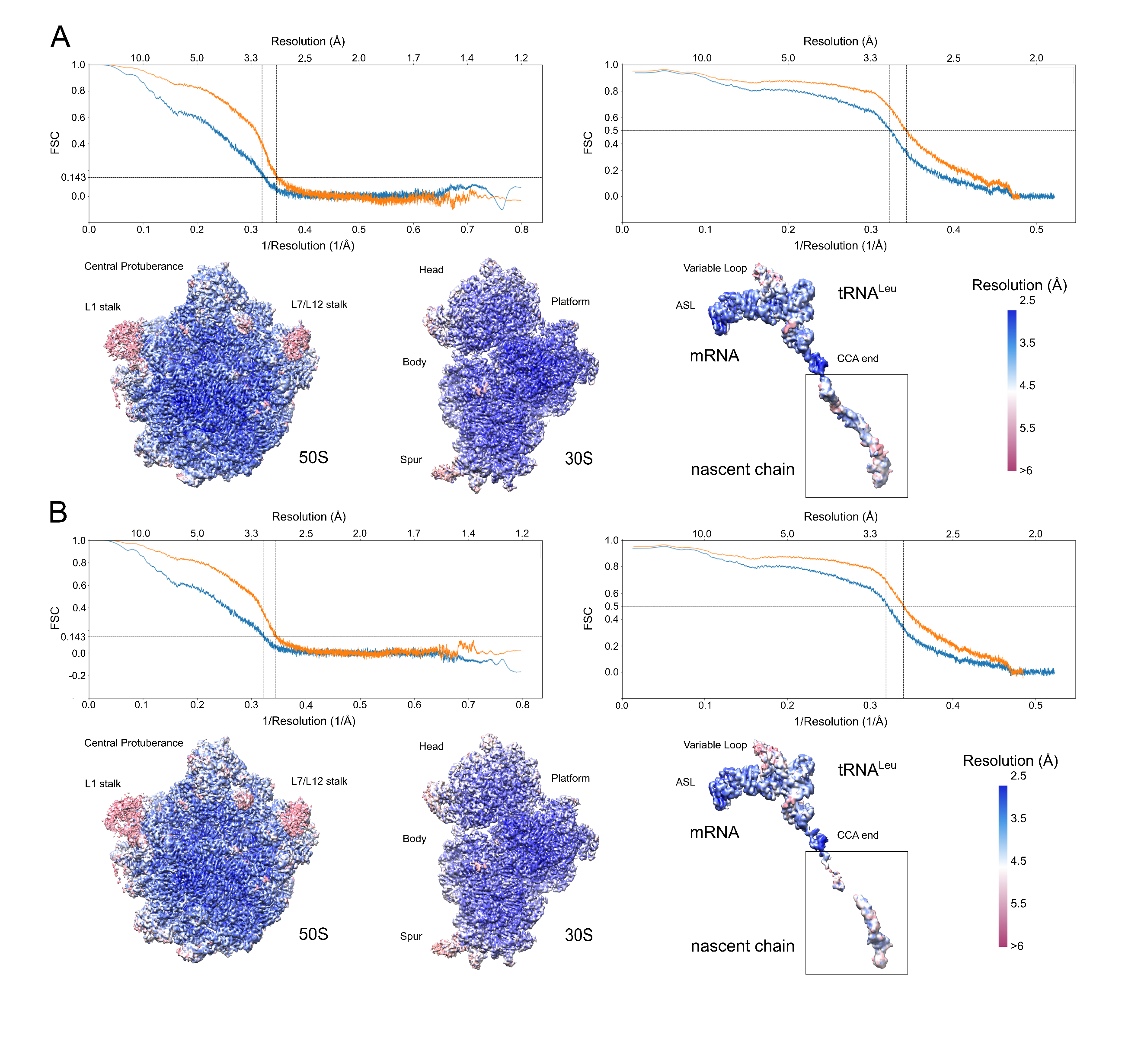


**Figure S9.** **FSC curves and the local resolution for the reconstructed cryoEM maps.** (A) CspA70-1. (B) CspA70-2. The FSC (Fourier Shell Correlation) curve for each of the three subsets is shown as upper panels, the curve for the final model *versus* the original, unsharpened map to the right. The 0.143 and 0.5 cutoffs in the FSC are highlighted. The soft mask used (masked FSC curves, orange) is calculated using the atomic model as previously described (Afonine et al., 2018). The plots were generated by the Phenix evaluation routine mtriage. Lower panels show the local resolution with scale bar providing color scale with resolution in Å. When compared to the CspA27 ensembles, the local resolution for the nascent chain is generally lower due to broader conformational fluctuations.


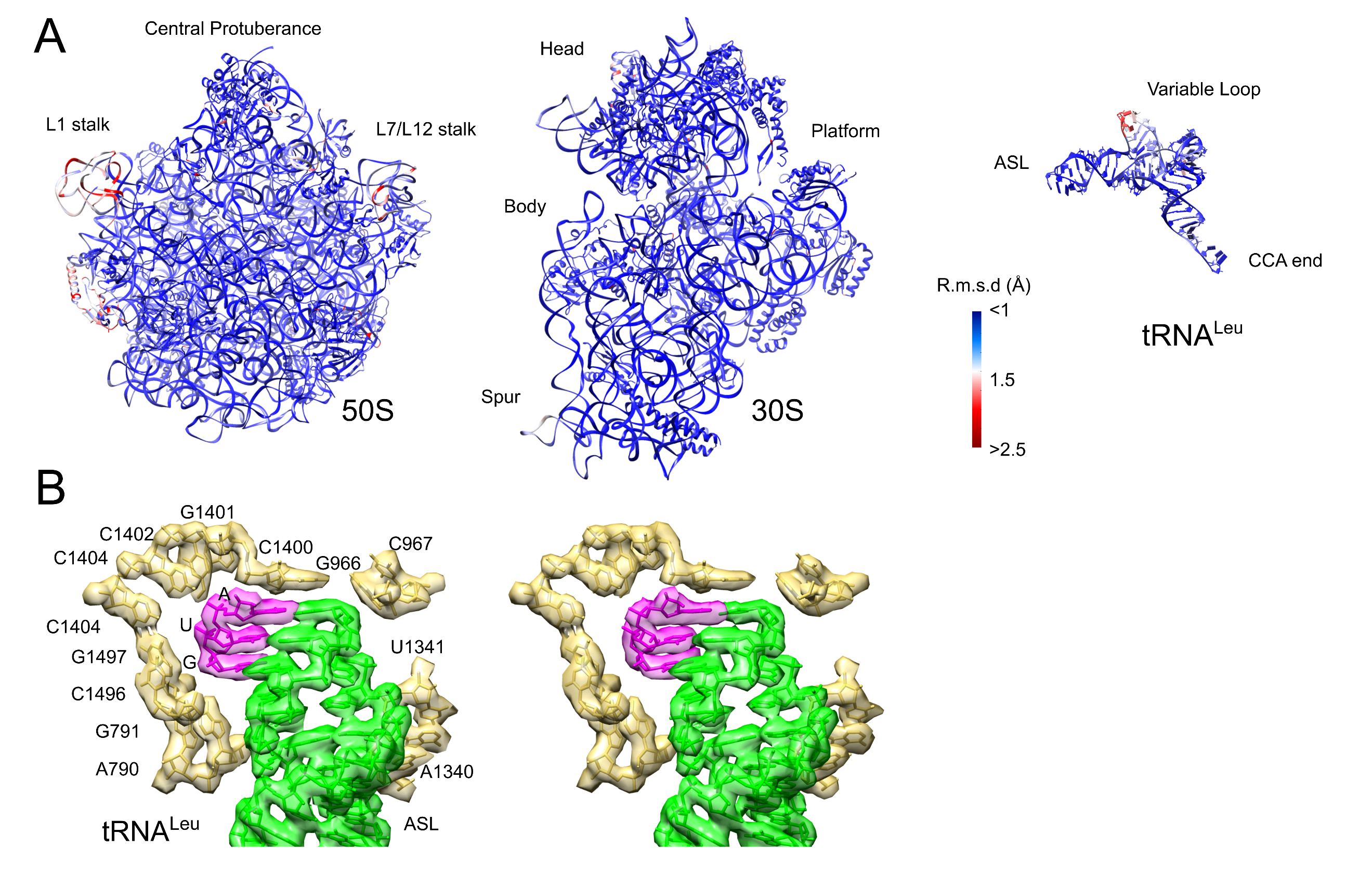


**Figure S10. CspA-FL, structure comparison.** (A) Superposition of CspA70-1 and -2 shows a root mean square deviation (rmsd) <1.5 Å for most atoms**.** Overall, no large-scale inter or intra-subunit rearrangements are observed when comparing the structures. Local variations are again ascribed to flexible ribosomal domains, as well as the variable of loop of the tRNA^Leu^ . Scale bar shows color scale with resolution in Å. (B) Close-up view of the codon-anticodon duplexes for CspA70-1 (left) and CspA70-2 (right) showing the framework of interactions at the tRNA anticodon stem-loop region.


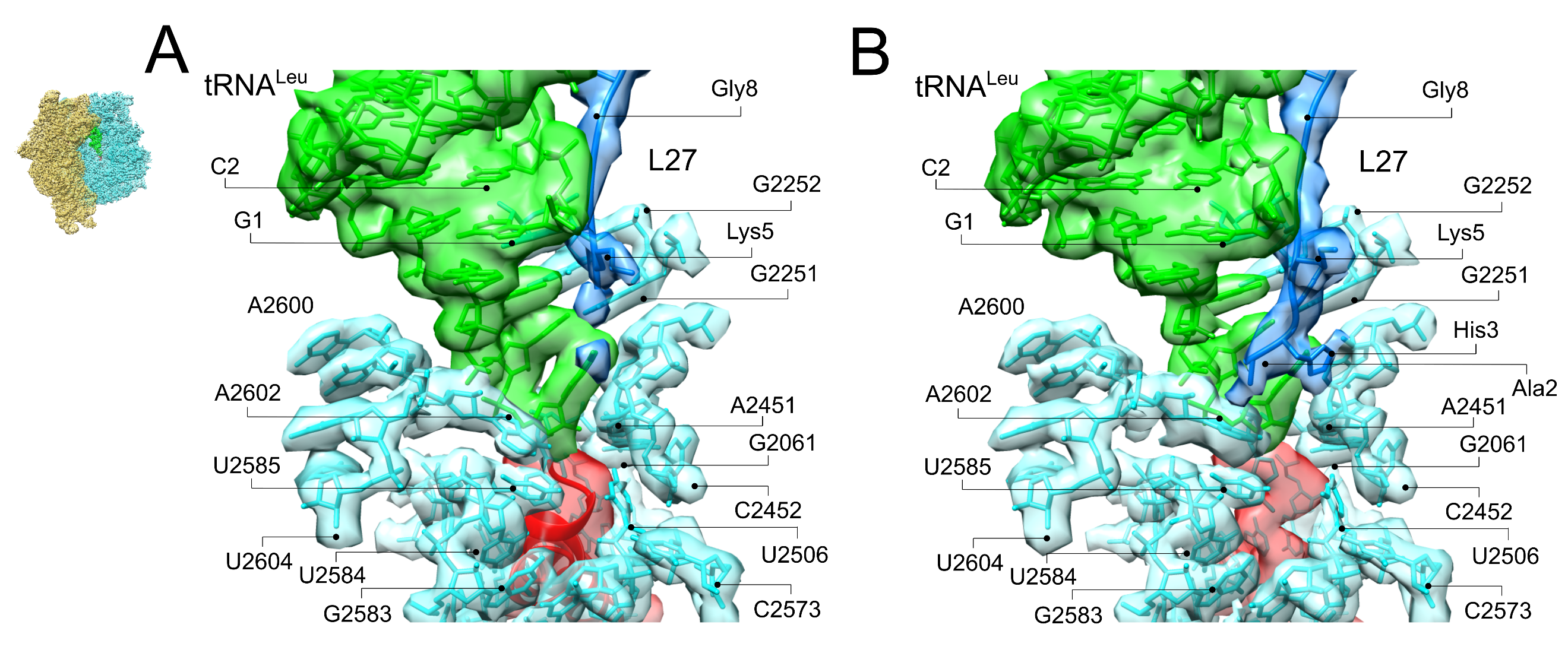


**Figure S11. CspA70,** **PTC conformation.** Close-up views of the PTC for (A) CspA70-1 and (B) CspA70-2. The cryo-EM density is presented in transparent red (peptide), green (tRNA), cyan (23S rRNA) and blue (ribosomal protein L27). The L27-2602 contact can be visualized when the map is contoured at around 1.7σ level. Inset shows the overall structure and orientation of the ribosome.


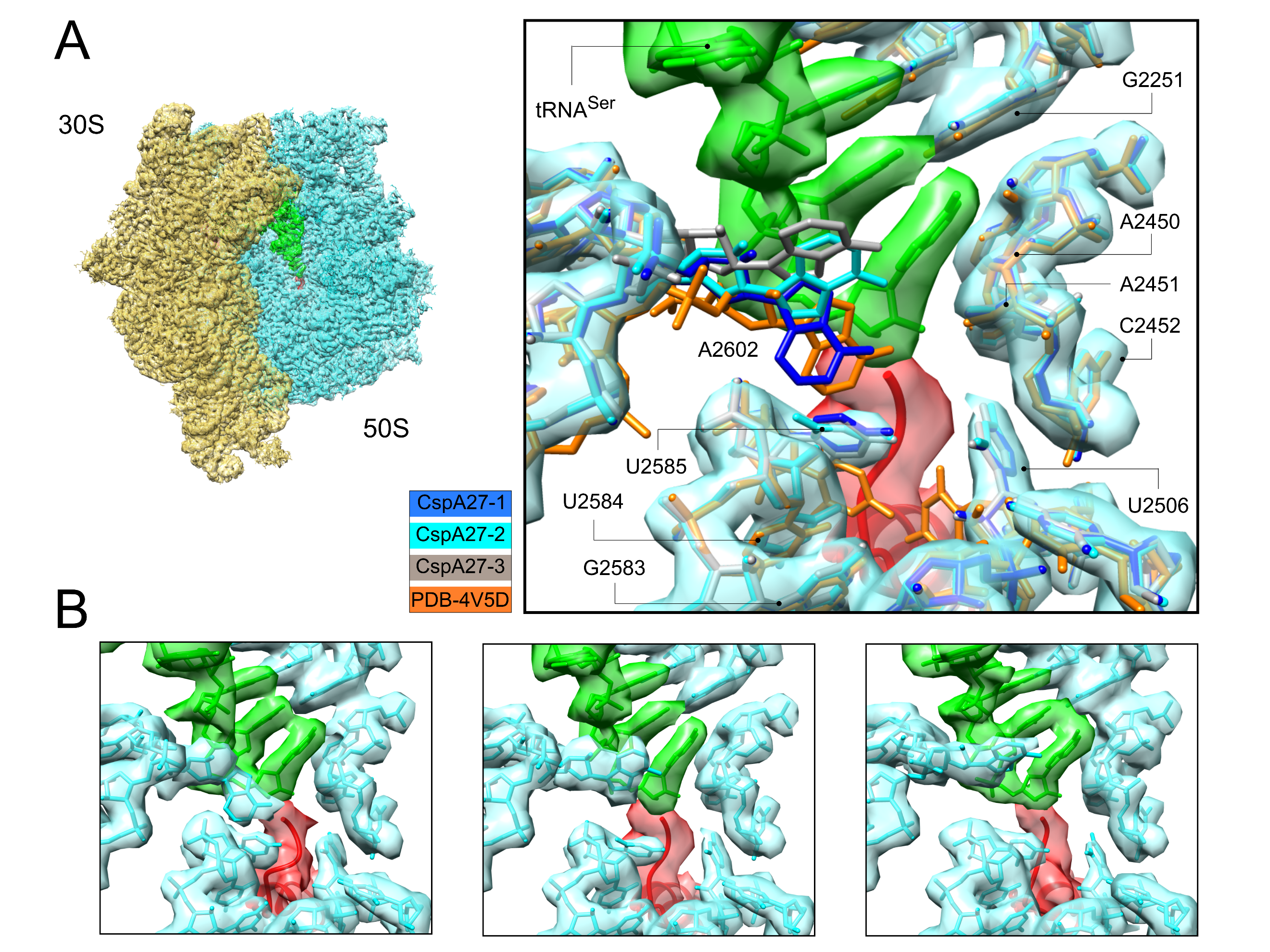


**Figure S12**. **CspA27,** **PTC conformation**. (A) Close-up view of superimposed PTC nucleotides for CspA27-1 (dark blue), CspA27-2 (cyan), CspA27-3 (grey) and induced state elongation complex (PDB 4V5D (Voorhees et al., 2009), in orange). A-site accommodation is accompanied by reorientation of nucleotides U2584, U2585 and U2506 towards the catalytically active or induce state conformation, which unlocks the PTC in order to expose the ester bond; during this transition, A2602 also shits towards the A site and helps in proper positioning of the incoming aa-tRNA (Schmeing et al., 2005, Voorhees et al., 2009). In extended conformation 1, partial shift of A2602 towards the A site is observed even when the A site is empty. The cryo-EM density corresponding to CspA27-2 is presented in transparent red (peptide), green (tRNA) and cyan (23S rRNA). The color scheme for comparison between structures is shown graphically. The density corresponding to A2602 was computationally removed for clarity. Inset shows the overall structure and orientation of the ribosome. (B) From left to right, PTC conformation in CspA27 conformations 1 to 3. Nucleotide A2602 shows weaker density and is displayed at lower contour level than the surrounding residues (contoured at ~1.5σ, instead of ~4 σ).

**APPENDIX TABLES**

**Table S1. Fitting of translation kinetics using a delay-exponential function.**

| Fitting parameter | Best-fit values | SEM (%) | 95% confidence interval |
| --- | --- | --- | --- |
| CspaA19 |  |  |  |
| Plateau | 0.99 | 1 | 0.968 to 1.007 |
| τ delay (s) | 0.68 | 12 | 0.507 to 0.826 |
| τ_exp_ (s) | 2.97 | 5 | 2.683 to 3.300 |
| lifetime τ (s) | 3.65 | 5 |  |
| Translation rate (aa/s) | 5.21 | 5 |  |
| R^2^ | 0.98 |  |  |
|  |  |  |  |
| CspA27 |  |  |  |
| Plateau | 1.23 | 5 | 1.120 to 1.381 |
| τ delay (s) | 4.65 | 4 | 4.260 to 4.960 |
| τ_exp_ (s) | 8.09 | 12 | 6.59 to 10.33 |
| lifetime τ (s) | 12.74 | 8 |  |
| Translation rate (aa/s) | 2.12 | 8 |  |
| R^2^ | 0.98 |  |  |
|  |  |  |  |
| CspA70 |  |  |  |
| Plateau | 1.02 | 2 | 0.987 to 1.057 |
| τ delay (s) | 9.67 | 2 | 9.307 to 10.05 |
| τ_exp_ (s) | 6.57 | 7 | 5.825 to 7.452 |
| lifetime τ (s) | 16.23 | 3 |  |
| Translation rate (aa/s) | 4.31 | 3 |  |
| R^2^ | 0.99 |  |  |

**Table S2: Fitting of CspA PET efficiency.** Fitting functions indicated within the table.

| Fitting parameter | Best-fit values | SEM (%) | 95% confidence interval |
| --- | --- | --- | --- |
| CspA14 PET |  |  |  |
| Plateau + 1 exponent |  |  |  |
| Plateau | 0.68 | 0.2 | 0.674 to 0.681 |
| τ delay (s) | 1.69 | 0.4 | 1.679 to 1.707 |
| τ_exp_ (s) | 0.84 | 2 | 0.815 to 0.867 |
| lifetime τ (s) | 2.53 | 1 |  |
| R^2^ | 0.99 |  |  |
|  |  |  |  |
| CspA19 PET |  |  |  |
| Plateau + 2 exponents |  |  |  |
| τ delay (s) | 1.75 | 0.3 | 1.742 to 1.765 |
| k_1_ (s^-1^) | 1.18 | 15 | 0.823 to 1.543 |
| τ_1_ (s) | 0.85 | 15 |  |
| lifetime τ_1_ (s) | 2.60 | 5 |  |
| k_2_ (s^-1^) | 0.81 | 19 | 0.502 to 1.114 |
| τ_2_ (s) | 1.24 | 19 |  |
| lifetime τ_2_ (s) | 3.84 | 8 |  |
| R^2^ | 0.99 |  |  |
|  |  |  |  |
| CspA27 PET |  |  |  |
| plateau + 2 exponents |  |  |  |
| τ delay (s) | 1.36 | 0.3 | 1.351 to 1.368 |
| k_1_ (s^-1^) | 1.68 | 9 | 1.393 to 1.965 |
| τ_1_ (s) | 0.60 | 9 |  |
| lifetime τ_1_ (s) | 1.95 | 3 |  |
| k_2_ (s^-1^) | 0.95 | 10 | 0.766 to 1.126 |
| τ_2_ (s) | 1.06 | 10 |  |
| lifetime τ_2_ (s) | 2.42 | 4 |  |
| R^2^ | 0.98 |  |  |

**Table S3. CryoEM data and model statistics**. Models were validated using the tools for analysis and validation integrated in the Phenix software suite (Afonine et al., 2018).

| **Data collection/processing** | **CspA27-1** | **CspA27-1** | **CspA27-3** | **CspA70-1** | **CspA70-2** |
| --- | --- | --- | --- | --- | --- |
| Voltage (kV) | 300 | 300 | 300 | 300 | 300 |
| Electron exposure (e–/Å^2^) | 55 | 55 | 55 | 55 | 55 |
| Defocus range (μm) | 0,5-2.2 | 0,5-2.2 | 0,5-2.2 | 0,5-2.2 | 0,5-2.2 |
| Pixel size (Å) | 1.07 | 1.07 | 1.07 | 1.07 | 1.07 |
| Symmetry imposed | C1 | C1 | C1 | C1 | C1 |
| Initial particle images | 1.404.270 | 1.404.270 | 1.404.270 | 814.221 | 814.221 |
| Final particle images | 35.573 | 44.182 | 15.459 | 26.765 | 23.782 |
| Map resolution (Å)  (FSC threshold 0.143) | 3.05 | 3.00 | 3.19 | 2.91 | 2.93 |
| **Model building** |  |  |  |  |  |
| Initial model used (PDB id) | 6ore | 6ore | 6ore | 6ore | 6ore |
| Model vs. Data fit (CC)  Model composition  Chains  Non-hydrogen atoms  Residues: Protein  Residues: Nucleotide | 0.88  55  145.149  5.738  4.651 | 0.88  55  145.152  5.738  4.651 | 0.86  55  145.136  5.738  4.651 | 0.86  55  145.377  5.778  4.648 | 0.86  55  145.186  5.754  4.648 |
| Ligands: ZN | 2 | 2 | 2 | 2 | 2 |
| Ligands: MG | 413 | 416 | 400 | 394 | 422 |
| Ramachandran plot (%)  Outliers  Allowed | 0.18  3.73 | 0.16  3.69 | 0.16  3.64 | 0.18  3.61 | 0.21  3.61 |
| Favored | 96.10 | 96.15 | 96.20 | 96.21 | 96.18 |
| R.m.s. deviations  Bond lengths (Å)  Bond angles (°)  Clash score  MolProbity score  Rotamer outliers  **C**β outliers (%) | 0.010  1.018  7.21  1.66  0.38  0.02 | 0.017  1.212  7.82  1.69  0.21  0.0 | 0.011  1.070  8.49  1.72  0.21  0.00 | 0.009  1.076  7.05  1.64  0.19  0.00 | 0.016  1.269  7.84  1.69  0.23  0.00 |

**Table S4. Fitting of PET-FCS autocorrelation curves using Equation 1 (Method Details).**

| Fitting parameter | Best-fit  values | SEM (%) | 95% confidence interval |  |
| --- | --- | --- | --- | --- |
| CspA70 W11 |  |  |  | |
| c_1_ | 0.20 | 3 | 0.186 to 0.223 | |
| k_1_ (s^-1^) | 3053495 | 6 | 2561605 to 3659696 |  |
| τ_1_ (1/k_1_) (μs) | 0.33 | 6 |  |  |
| c_2_ | 0.55 | 1 | 0.528 to 0.563 |  |
| k_2_ (s^-1^) | 416489 | 2 | 391384 to 440389 |  |
| τ_2_ (1/k_2_) (μs) | 2.40 | 2 |  |  |
| F | 0.20 | 1 | 0.194 to 0.204 |  |
| k_f_ (s^-1^) | 19664 | 3 | 17950 to 21509 |  |
| τ_f_ (1/k_f_) (μs) | 50.9 | 3 |  |  |
| N | 0.96 | 0.3 | 0.954 to 0.967 |  |
| k_d_ (s^-1^) | 845.2 | 1 | 830.7 to 859.6 |  |
| τ_d_ (1/k_d_) (ms) | 1.18 | 1 |  |  |
| R^2^ | 0.99 |  |  |  |
|  |  |  |  |  |
| CspA 70 W11L |  |  |  |  |
| c_1_ | 0.20 | 4 | 0.176 to 0.217 |  |
| k_1_ (s^-1^) | 2501503 | 6 | 2137422 to 2945086 |  |
| τ_1_ (1/k_1_) (μs) | 0.40 | 6 |  |  |
| c_2_ | 0.47 | 2 | 0.445 to 0.483 |  |
| k_2_ (s^-1^) | 401610 | 2 | 375509 to 426615 |  |
| τ_2_ (1/k_2_) (μs) | 2.49 | 2 |  |  |
| F | 0.14 | 2 | 0.133 to 0.143 |  |
| k_f_ (s^-1^) | 17344 | 4 | 15463 to 19428 |  |
| τ_f_ (1/k_f_) (μs) | 57.7 | 4 |  |  |
| N | 0.96 | 0.2 | 0.951 to 0.961 |  |
| k_d_ (s^-1^) | 794 | 1 | 782.5 to 805.3 |  |
| τ_d_ (1/k_d_) (ms) | 1.26 | 1 |  |  |
| R^2^ | 0.99 |  |  |  |

**Table S5. Amino acid and DNA sequence of CspA variants, green – native W11, red – mutated aa residues).**

| Construct | Amino acid sequence | DNA sequence 5’ to 3’ |
| --- | --- | --- |
| WT | MSGKMTGIVKWFNADKGFGFITPDDGSKDVFVHFSAIQNDGYKSLDEGQKVSFTIESGAKGPAAGNVTSL | ATGTCCGGTAAAATGACTGGTATCGTAAAATGGTTCAACGCTGACAAAGGCTTCGGCTTCATCACTCCTGACGATGGCTCTAAAGATGTGTTCGTACACTTCTCTGCTATCCAGAACGATGGTTACAAATCTCTGGACGAAGGTCAGAAAGTGTCCTTCACCATCGAAAGCGGCGCTAAAGGCCCGGCAGCTGGTAACGTAACCAGCCTGTAA |
| W11L | MSGKMTGIVKLFNADKGFGFITPDDGSKDVFVHFSAIQNDGYKSLDEGQKVSFTIESGAKGPAAGNVTSL | ATGTCCGGTAAAATGACTGGTATCGTAAAATTGTTCAACGCTGACAAAGGCTTCGGCTTCATCACTCCTGACGATGGCTCTAAAGATGTGTTCGTACACTTCTCTGCTATCCAGAACGATGGTTACAAATCTCTGGACGAAGGTCAGAAAGTGTCCTTCACCATCGAAAGCGGCGCTAAAGGCCCGGCAGCTGGTAACGTAACCAGCCTGTAA |
| F20W | MSGKMTGIVKLFNADKGFGWITPDDGSKDVFVHFSAIQNDGYKSLDEGQKVSFTIESGAKGPAAGNVTSL | ATGTCCGGTAAAATGACTGGTATCGTAAAATTGTTCAACGCTGACAAAGGCTTCGGCTGGATCACTCCTGACGATGGCTCTAAAGATGTGTTCGTACACTTCTCTGCTATCCAGAACGATGGTTACAAATCTCTGGACGAAGGTCAGAAAGTGTCCTTCACCATCGAAAGCGGCGCTAAAGGCCCGGCAGCTGGTAACGTAACCAGCCTGTAA |
| WT for expression | MSGKMTGIVKWFNADKGFGFITPDDGSKDVFVHFSAIQNDGYKSLDEGQKVSFTIESGAKGPAAGNVTSLENLYFQGHHHHHH | ATGTCCGGTAAAATGACTGGTATCGTAAAATGGTTCAACGCTGACAAAGGCTTCGGCTTCATCACTCCTGACGATGGCTCTAAAGATGTGTTCGTACACTTCTCTGCTATCCAGAACGATGGTTACAAATCTCTGGACGAAGGTCAGAAAGTGTCCTTCACCATCGAAAGCGGCGCTAAAGGCCCGGCAGCTGGTAACGTAACCAGCCTGGAGAATCTTTATTTTCAGGGCCACCACCACCACCACCACTGA |

**Table S6. Primer sequences used to generate mRNA templates of varying CspA lengths.**

| Primer for: | Sequence |
| --- | --- |
| FW T7 | TAATACGACTCACTATAGGG |
| RV CspA 14 W11 (WT) | AGCGTTGAACCATTTTACGATACC |
| RV CspA 14 W11L | AGCGTTGAACAATTTTACGATACCAGTC |
| RV CspA 19 | GCCGAAGCCTTTGTCAGCGTT |
| RV CspA 27 | AGAGCCATCGTCAGGAGTGATGAAG |
| RV CspA FL | CAGGCTGGTTACGTTACCAGCTGC |

**Table S7. CspA FPA constructs N to C terminus.** Numbers in superscript correspond to Table S8 primers, Figure 3B and Figure S2C indicating length of CspA in truncations.

| CspA 1-70 aa | SecM 71-87 aa | LepB 88-110 aa |
| --- | --- | --- |
| MSG^3^ KM^5^ TG^7^ IV^9^ KW^11^ FN^13^ AD^15^ KG^17^ FG^19^ FI^21^ TP^23^ DD^25^ GS^27^ KD^29^ VF^31^ VH^33^ FS^35^ AI^37^ QN^39^ DG^41^ YK^43^ SL^45^ DE^47^ GQ^49^ KV^51^ SF^53^ TI^55^ ES^57^ GA^59^ KG^61^ PA^63^ AG^65^ NV^67^ TS^69^ L^70^ | FSTPVWISQA QGIRAGP | GSSDKQEGEW PTGLRLSRIG GIH |

**Table S8. Primers used to generate CspA plasmid variants for FPA**. FW – forward primer; RV – reverse primer; numbers indicate length of CspA sequence from N-terminus in amino acids.

| Primer | Sequence |
| --- | --- |
| FW T7 | TTCAGCACGCCCGTCTGGATAAG |
| RV 5 | CATTTTACCGGACATATGTATATCTCCTTC |
| RV 7 | ACCAGTCATTTTACCGGACATATGTATATCTCC |
| RV 9 | TACGATACCAGTCATTTTACCGGACATATG |
| RV 11 | CCATTTTACGATACCAGTCATTTTACCG |
| RV 13 | GTTGAACCATTTTACGATACCAGTCATTTTAC |
| RV 15 | GTCAGCGTTGAACCATTTTACGATACC |
| RV 17 | GCCTTTGTCAGCGTTGAACCATTT |
| RV 19 | GCCGAAGCCTTTGTCAGCG |
| RV 21 | GATGAAGCCGAAGCCTTTGTCAGC |
| RV 23 | AGGAGTGATGAAGCCGAAGCCTTTG |
| RV 25 | ATCGTCAGGAGTGATGAAGCCGAAG |
| RV 27 | AGAGCCATCGTCAGGAGTGATGAAG |
| RV 29 | ATCTTTAGAGCCATCGTCAGGAGTGATG |
| RV 33 | GTGTACGAACACATCTTTAGAGCCATCG |
| RV 35 | AGAGAAGTGTACGAACACATCTTTAGAGCC |
| RV 37 | GATAGCAGAGAAGTGTACGAACACATCTTTAG |
| RV 39 | GTTCTGGATAGCAGAGAAGTGTACGAACAC |
| RV 41 | ACCATCGTTCTGGATAGCAGAGAAGTG |
| RV 43 | TTTGTAACCATCGTTCTGGATAGCAGAG |
| RV 45 | CAGAGATTTGTAACCATCGTTCTGGATAGC |
| RV 47 | TTCGTCCAGAGATTTGTAACCATCG |
| RV 49 | CTGACCTTCGTCCAGAGATTTGTAACC |
| RV 51 | CACTTTCTGACCTTCGTCCAGAGATTTG |
| RV 53 | GAAGGACACTTTCTGACCTTCGTCC |
| RV 55 | GATGGTGAAGGACACTTTCTGACCTTC |
| RV 57 | GCTTTCGATGGTGAAGGACACTTTC |
| RV 59 | AGCGCCGCTTTCGATGGTG |
| RV 61 | GCCTTTAGCGCCGCTTTCG |
| RV 63 | TGCCGGGCCTTTAGCGCC |
| RV 65 | ACCAGCTGCCGGGCCTTTAG |
| RV 67 | TACGTTACCAGCTGCCGGGC |
| RV 69 | GCTGGTTACGTTACCAGCTGCCG |

**APPENDIX REFERENCES**

Afonine PV, Klaholz BP, Moriarty NW, Poon BK, Sobolev OV, Terwilliger TC, Adams PD, Urzhumtsev A (2018) New tools for the analysis and validation of cryo-EM maps and atomic models. *Acta Crystallogr D Struct Biol* 74: 814-840

Schmeing TM, Huang KS, Strobel SA, Steitz TA (2005) An induced-fit mechanism to promote peptide bond formation and exclude hydrolysis of peptidyl-tRNA. *Nature* 438: 520-4

Voorhees RM, Weixlbaumer A, Loakes D, Kelley AC, Ramakrishnan V (2009) Insights into substrate stabilization from snapshots of the peptidyl transferase center of the intact 70S ribosome. *Nat Struct Mol Biol* 16: 528-33
