# Supplementary material for: A switch from α‐helical to β‐strand conformation during co‐translational protein folding
Source: EMBO J. 2022 Jan 7;41(4):e109175. doi: 10.15252/embj.2021109175 (PMC8844987; doi:10.15252/embj.2021109175)
Supplement: Supplementary file 5 — Movie EV4 [file EMBJ-41-e109175-s008.zip › Movie_EV4_legend.docx]

**EXPANDED VIEW (large files) LEGENDS**

**Movies EV4, EV5. Conformational transitions for CspA27.** The atomic models of nascent chain and large ribosomal proteins L4 and L22 are depicted using ribbon representations (red and blue, respectively). Movie EV4: transition from conformations 1 and 2, showing how the helix begins to break up at the N-terminus and then gradually unwinds towards the C-terminus. Movie EV5: transition from conformations 2 and 3, showing how the helix starts to break up in the middle part and ends up forming two smaller helical modules. The access route to the different metastable conformation is correlated with rearrangements in several arginine residues, which by interaction with certain residues or by intercalation into the helical grooves, might stabilize/destabilize the different conformations. These transient changes might be part of a dynamic and flexible gate that governs the transition from and into the different metastable conformations. The trajectories between conformations were created by morphing structures using Chimera (Pettersen et al., 2004).

Pettersen EF, Goddard TD, Huang CC, Couch GS, Greenblatt DM, Meng EC, Ferrin TE (2004) UCSF Chimera--a visualization system for exploratory research and analysis. *J Comput Chem* 25: 1605-12
