# Supplementary material for: A switch from α‐helical to β‐strand conformation during co‐translational protein folding
Source: EMBO J. 2022 Jan 7;41(4):e109175. doi: 10.15252/embj.2021109175 (PMC8844987; doi:10.15252/embj.2021109175)
Supplement: Supplementary file 7 — Movie EV6 [file EMBJ-41-e109175-s007.zip › Movie_EV6_legend.docx]

**EXPANDED VIEW (large files) LEGENDS**

**Movie EV6. CspA70-1, the peptide and ribosomal tunnel.** Structure visualization for conformation 1. The cryo-EM density is presented in transparent red (peptide), green (tRNA) and yellow (30S subunit). The 50S subunit is removed for clarity. Cryo-EM densities showing the large ribosomal proteins L4, L22, L23 and L24, as well as the 23S rRNA nucleotides that decorate the tunnel and exit port are shown in blue and cyan, respectively. The atomic models are depicted using ribbon representations. The movies were created using Chimera (Pettersen et al., 2004).

Pettersen EF, Goddard TD, Huang CC, Couch GS, Greenblatt DM, Meng EC, Ferrin TE (2004) UCSF Chimera--a visualization system for exploratory research and analysis. *J Comput Chem* 25: 1605-12
