# Supplementary material for: A switch from α‐helical to β‐strand conformation during co‐translational protein folding
Source: EMBO J. 2022 Jan 7;41(4):e109175. doi: 10.15252/embj.2021109175 (PMC8844987; doi:10.15252/embj.2021109175)
Supplement: Supplementary file 9 — Movie EV8 [file EMBJ-41-e109175-s003.zip › Movie_EV8_legend.docx]

**EXPANDED VIEW (large files) LEGENDS**

**Movie EV8. Conformational transition for CspA70 in the exit port.** The trajectory between conformations was created by morphing structures using Chimera (Pettersen et al., 2004) and suggests a plausible conformational transition for the N-terminal nascent chain region. Cryo-EM densities showing the large ribosomal proteins L22, L23, L24 and L29, as well as the 23S rRNA nucleotides that decorate the exit port are shown in blue/cyan, the remaining 50S structure in grey.

Pettersen EF, Goddard TD, Huang CC, Couch GS, Greenblatt DM, Meng EC, Ferrin TE (2004) UCSF Chimera--a visualization system for exploratory research and analysis. *J Comput Chem* 25: 1605-12
