# Supplementary material for: A switch from α‐helical to β‐strand conformation during co‐translational protein folding
Source: EMBO J. 2022 Jan 7;41(4):e109175. doi: 10.15252/embj.2021109175 (PMC8844987; doi:10.15252/embj.2021109175)
Supplement: Supplementary file 10 — Movie EV9 [file EMBJ-41-e109175-s005.zip › Movie_EV9_legend.docx]

**EXPANDED VIEW (large files) LEGENDS**

**Movie EV9. Overview of CspA cotranslational folding (CspA-27).** Cryo-EM densities are presented in transparent red (peptide), green (tRNA), yellow (30S subunit) and cyan (50S subunit). Cryo-EM densities showing the large ribosomal proteins L4, L22, L23, L24, L27 and L29 are shown in blue; the 23S rRNA nucleotides that decorate the PTC, ribosomal tunnel and exit port are shown in cyan. The induced state elongation complex (PDB 4V5D (Voorhees et al., 2009)) is shown in orange. The atomic models are depicted using ribbon representations*.* The movies were created using Chimera (Pettersen et al., 2004).

Voorhees RM, Weixlbaumer A, Loakes D, Kelley AC, Ramakrishnan V (2009) Insights into substrate stabilization from snapshots of the peptidyl transferase center of the intact 70S ribosome. *Nat Struct Mol Biol* 16: 528-33

Pettersen EF, Goddard TD, Huang CC, Couch GS, Greenblatt DM, Meng EC, Ferrin TE (2004) UCSF Chimera--a visualization system for exploratory research and analysis. *J Comput Chem* 25: 1605-12
