# Supplementary material for: A switch from α‐helical to β‐strand conformation during co‐translational protein folding
Source: EMBO J. 2022 Jan 7;41(4):e109175. doi: 10.15252/embj.2021109175 (PMC8844987; doi:10.15252/embj.2021109175)
Supplement: Supplementary file 11 — Movie EV10 [file EMBJ-41-e109175-s002.zip › Movie_EV10_legend.docx]

**EXPANDED VIEW (large files) LEGENDS**

**Movie EV10. Overview of CspA cotranslational folding (CspA-70).** Cryo-EM densities are presented in transparent red (peptide), green (tRNA), yellow (30S subunit) and cyan (50S subunit). Cryo-EM densities showing the large ribosomal proteins L4, L22, L23, L24, L27 and L29 are shown in blue; the 23S rRNA nucleotides that decorate the PTC, ribosomal tunnel and exit port are shown in cyan. RF1 (PDB-5J4D (Svidritskiy et al., 2016)) is shown in yellow. The atomic models are depicted using ribbon representations*.* The movies were created using Chimera (Pettersen et al., 2004).

Svidritskiy E, Madireddy R, Korostelev AA (2016) Structural Basis for Translation Termination on a Pseudouridylated Stop Codon. *J Mol Biol* 428: 2228-36

Pettersen EF, Goddard TD, Huang CC, Couch GS, Greenblatt DM, Meng EC, Ferrin TE (2004) UCSF Chimera--a visualization system for exploratory research and analysis. *J Comput Chem* 25: 1605-12
