# Supplementary figures and images for: A switch from α‐helical to β‐strand conformation during co‐translational protein folding
Source: EMBO J. 2022 Jan 7;41(4):e109175. doi: 10.15252/embj.2021109175 (PMC8844987; doi:10.15252/embj.2021109175)

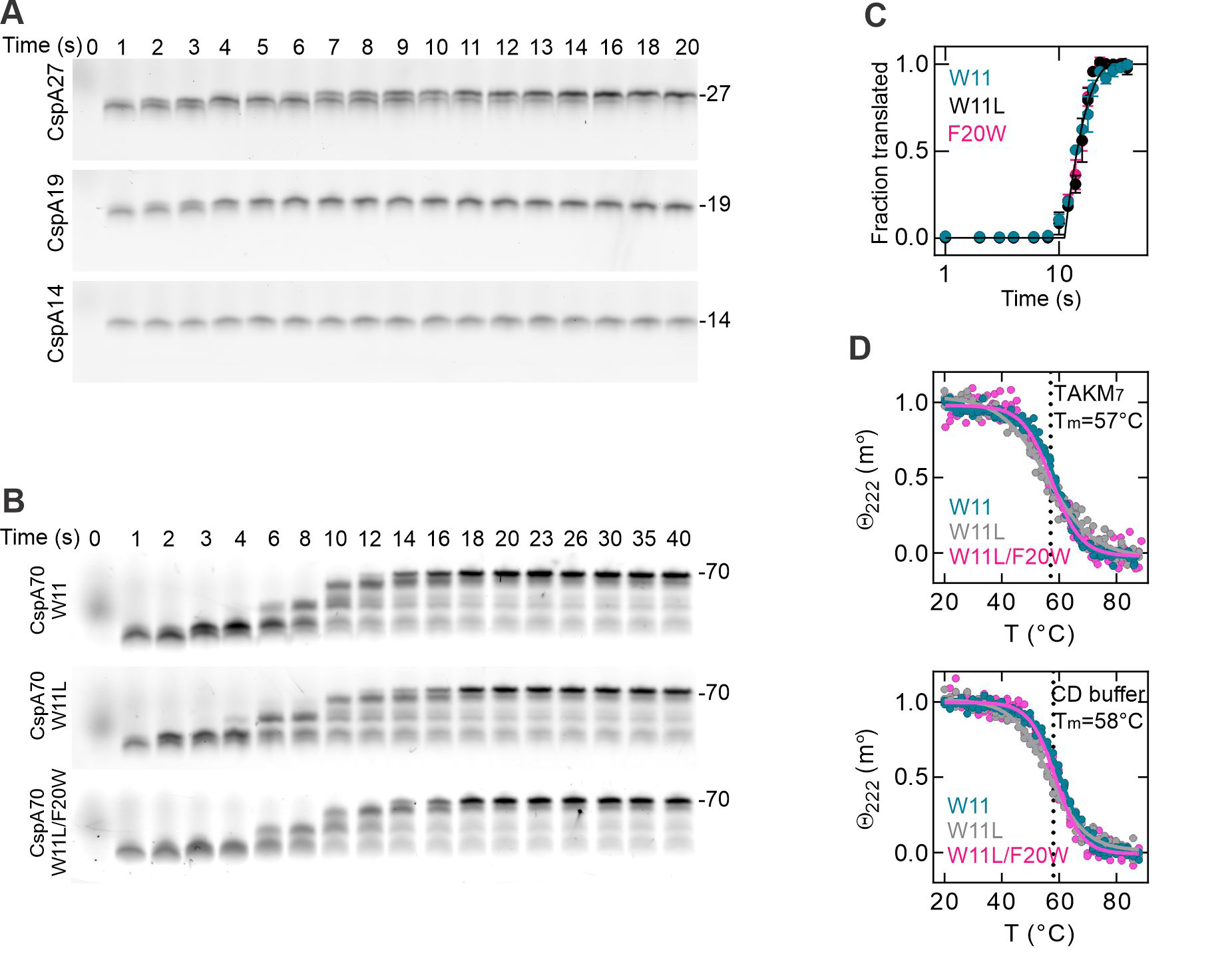

Supplement: Supplementary file 12 — Source Data for Appendix [file EMBJ-41-e109175-s011.zip › source_data_appendix_figS1/Sfig1.png]

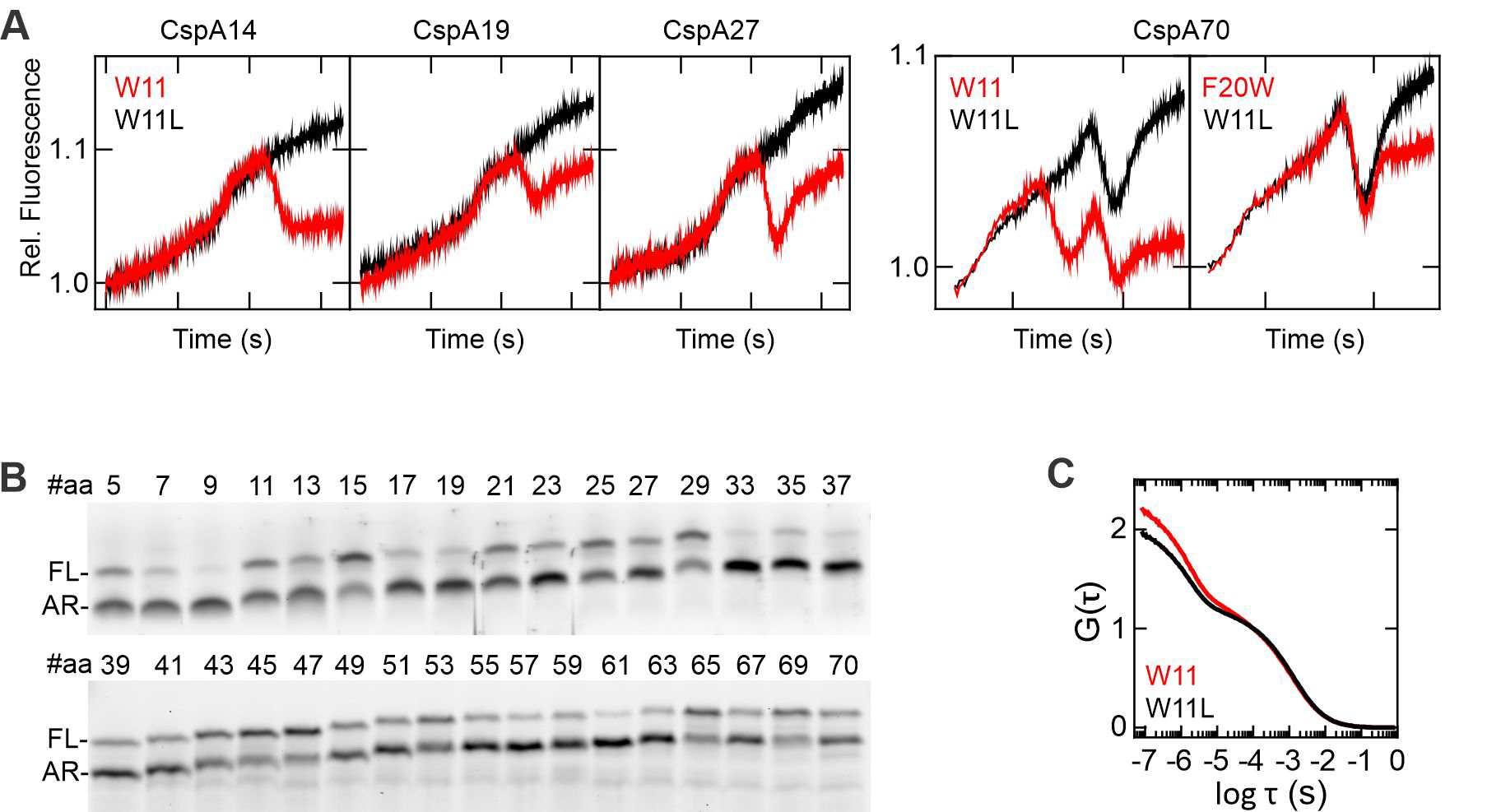

Supplement: Supplementary file 12 — Source Data for Appendix [file EMBJ-41-e109175-s011.zip › source_data_appendix_figS2/Sfig2.png]

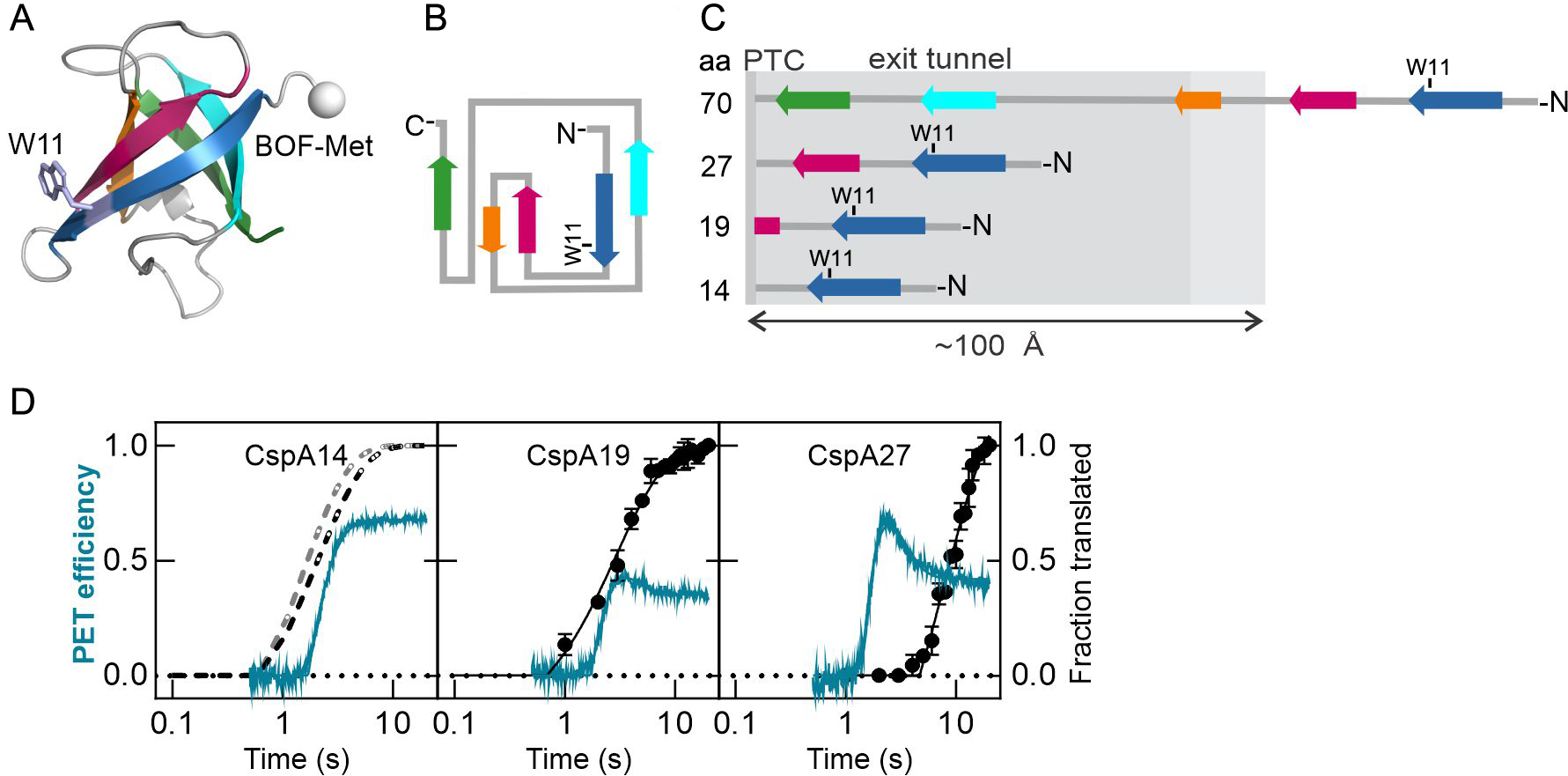

Supplement: Supplementary file 13 — Source Data for Figure 1 [file EMBJ-41-e109175-s004.zip › source_data_fig1/Fig1.png]

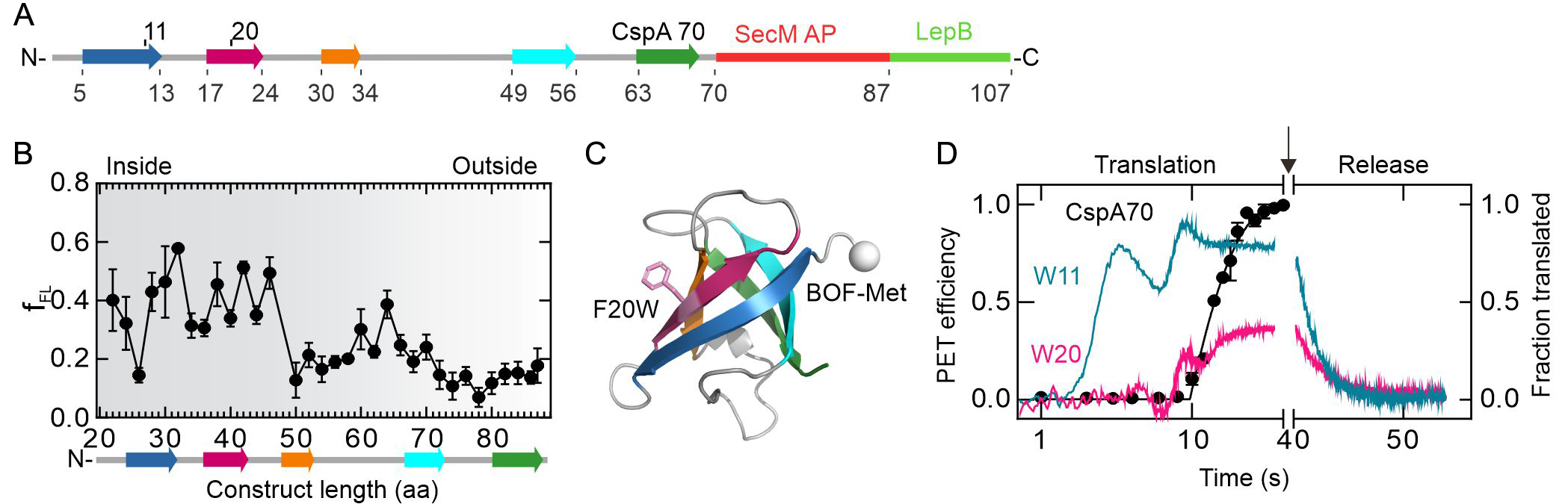

Supplement: Supplementary file 14 — Source Data for Figure 3 [file EMBJ-41-e109175-s010.zip › source_data_fig3/Fig3.png]
