# Supplementary material for: A switch from α‐helical to β‐strand conformation during co‐translational protein folding
Source: EMBO J. 2022 Jan 7;41(4):e109175. doi: 10.15252/embj.2021109175 (PMC8844987; doi:10.15252/embj.2021109175)
Supplement: Supplementary file 15 [file EMBJ-41-e109175-s012.pdf]

THE EMBO JOURNAL

A switch from α-helical to β-strand conformation during co-translational protein folding

Xabier Agirrezabala, Ekaterina Samatova, Meline Macher, Marija Liutkute, Manisankar Maiti, David Gil-Carton, Jiri Novacek, Mikel Valle, and Marina Rodnina
DOI: 10.15252/embj.2021109175

Corresponding author(s): Marina Rodnina (rodnina@mpibpc.mpg.de) , Xabier Agirrezabala (xagirrezabala@cicbiogune.es)

Review Timeline:

Submission Date:	10th Jul 21
Editorial Decision:	24th Aug 21
Appeal Received:	26th Aug 21
Editorial Decision:	2nd Sep 21
Revision Received:	1st Oct 21
Editorial Decision:	22nd Nov 21
Revision Received:	30th Nov 21
Accepted:	6th Dec 21

Editor: Stefanie Boehm

Transaction Report:

(Note: With the exception of the correction of typographical or spelling errors that could be a source of ambiguity, letters and reports are not edited. Depending on transfer agreements, referee reports obtained elsewhere may or may not be included in this compilation. Referee reports are anonymous unless the Referee chooses to sign their reports.)

Prof. Marina V. Rodnina
Max Planck Institute for Biophysical Chemistry
Department of Physical Biochemistry
Am Fassberg 11
Goettingen 37077
Germany

24th Aug 2021

Re: EMBOJ-2021-109175
Switching from α-helical to β-strand conformation upon cotranslational protein folding

Dear Prof. Rodnina,

Thank you for submitting your manuscript on folding of CspA inside the ribosome exit tunnel for consideration by The EMBO Journal. Please also excuse the delay in communicating this decision to you, which was due to delayed referee responses due to the summer holiday period, as well as absences from the office. We have now however received three referee reports on your study, which are included below for your information. I have discussed the these reports and the manuscript with the other members of the editorial team. However, taking everything into consideration, I regret to say that we have decided that we unfortunately cannot offer to invite a revision for The EMBO Journal.

As you will see, all reviewers appreciate the experimental quality of the analysis and the novel cryo-EM structures. Yet, while we do recognize the additional insight into co-translational folding of CspA this provides, one of our main concerns is the overall advance for a broader audience, which is also a major point raised by referee #1 in particular. In addition to the more technical comments, referee #2 also agreed with this assessment when asked to comment on the other reports. As referee #1 indicates, we also find that the proposed link between nascent chain structure and PTC activity would be of additional interest, however at this stage, find that more mechanistic insight would be required. We realize that referee #3 is overall more positive in her/his assessment, but taking everything into consideration we have concluded that the study does not provide the degree of conceptual advance for a broader audience at the current time that would be required for publication in The EMBO Journal. Therefore, we unfortunately cannot offer to invite a revision at this stage.

I am sorry that I cannot be more positive on this occasion, but hope that you will nonetheless find the comments of our reviewers helpful. Thank you again for giving us the opportunity to consider your manuscript.

Kind regards,

Stefanie Boehm

Stefanie Boehm
Editor
The EMBO Journal

****

Referee #1:

The manuscript by Agirrezabala et al. investigates the conformations of short nascent chains of the cold shock protein CspA within the exit tunnel of the ribosome. Although the native structure of CspA is a small 5-strand beta-barrel, the authors show, using a combination of experimental techniques, that the CspA N-terminus adopts an alpha-helical conformation while 'high' in the tunnel (close to the peptidyl transferase center (PTC)). Once the nascent chain is sufficiently long as to reach the wider 'vestibule' portion near the end of the nascent chain exit tunnel, there is a conformational switch to a more native-like beta-hairpin conformation. Overall, this study, which combines a tour de force of kinetic PET measurements, cryoEM, force-profile measurements and other biochemical and biophysical analyses, represents a solid contribution to our still very sparse understanding of co-translational protein folding mechanisms. The experiments are well-constructed and controlled. However, previous studies from other groups, including von Heijne and Beckmann using force-profile and cryoEM ((2015 Cell Reports) and (2016) FEBS Lett.), and Johnson and Bernstein using FRET ((2004) Cell and (2006) Mol. Cell), have reported similar folding behavior for short nascent chains within the ribosome exit tunnel. For this reason, I expect the new insights from this

study will be primarily of interest to others in this immediate research area (protein synthesis and co-translational folding), rather than a broader audience.

I have only one major concern with this study, which is the authors' claim that "nascent polypeptide folding could locally regulate the elongation rate by modulating aa-tRNA accommodation" (final paragraph of main text) and "These data suggest a link between the nascent peptide conformation and the PTC activity, which may result in local attenuation of translation speed depending on the nascent chain folding" (Discussion). This is an intriguing claim but is unfortunately not supported by the results of this study, as it appears to be based only on an altered position for A2602 in the cryoEM structure of nascent CspA-27 at low contour level. Making such a mechanistic claim would require, at a minimum, demonstrating that the A2602 position is sensitive to the foldability of CspA-27, and that this A2602 position does affect PTC activity.

One minor point:
The abstract uses "configuration" to describe the differences in nascent chain conformation (not configuration).

Referee #2:

This study investigates folding of the beta-sheet protein CspA inside of the ribosomal exit tunnel. Nascent peptides typically interact with the tunnel walls, and formation of α-helices inside the tunnel has been reported for several proteins, including both types: soluble and membrane. This is explained by stability considerations, as α-helix is the most stable secondary structure. The current study illustrates that the same principle is also applied to a beta-sheet protein.

The study first shows three cryo-EM structures with a short CspA version, where the resolution for the nascent chain is 4-6 Å, allowing secondary structure determination, but not permitting residue tracing. The differences between the structures appear to be in the density inside the ribosomal tunnel, which is difficult to classify due to a disordered nature of the nascent peptide, and therefore all three classes probably represent a mixture of similar but slightly different partial folding. Interestingly, the comparison between the states shows changes in the orientation of positively charged Arg residues of proteins uL4 and uL22.

Next, previously established force profile approach was used to map the appearance of folding events by employing SecM stalling sequence. Specific folding events were detected, and the results were confirmed by photoinduced electron transfer method.
The manuscript then describes cryo-EM analysis of the full CspA length. Here two different classes are reported. Although the local resolution is not indicated, again they probably represent a mixture of partially folded nascent chains, and in one of the classes the nascent chain density is not continuous. While it is not explained why two classes were selected, and not any other number, the authors could identify somewhat distinct conformations of some of the ribosomal residues, although as mentioned in the text, a lower threshold of the map was needed for some cases due to the flexibility and mixed conformations.

The organisation of the manuscript is logical and convenient to follow, the authors also clearly describe the limitations of the study in the cryo-EM part, which is obviously a weak point of this work.
I have some technical comments and suggestions regarding the presentation of the study that the authors might want considering to improve the communication of their work.

Cryo-EM part:
1) The classification of the full CspA length, shown in Figure S8 would benefit from some clarification. A particular concern is that 2 classes contain similar number of particles (53%/47%), and therefore an additional indication is needed. Please describe the entire workflow and how the result was obtained, particularly what was the data when classification into 3 and 4 classes was performed. It is particularly important because for the short CspA version, a different classification into 3 classes was performed.

2) A table summarizing the cryo-EM data collection and processing parameters would help.

Model building part:
3) I went through the model, and it is generally ok. However, since this work illustrates conformational changes of single ribosomal residues, it is particularly important to pay attention to the quality of the model. The clash scores of 7-8 unfortunately raises unnecessary doubts in the model building. It would be a simple exercise in model building to improve the clashes, and I think for this type of work, the model needs to be sound, so that the findings are technically justified. Since the authors used Phenix for refinement, to make it easier, one way to start improving the quality of the model and taking care of the clashes is to generate hydrogens for refinement.

Figures:
Generally, the quality of the figures can be better. Although I understand that the authors try to keep minimal annotations, sometimes it is really difficult to follow. Here are some specific examples:

4) Figure 1: please extend the labeling to all the panels, so that it is easier to identify specific residues and compare between the states.

5) Figure 2: also here, please add labels.

6) Figure 4: please show panels A and B in the same representation, so that it is easier to compare. If I understand correctly, panel C is close-up of A, then it would be good to connect and show they are related.

7) Figure 5: please add labels, indicate in the figure what is shown, as well as the color scheme. It would make much easier to understand. Lower panel is close-up of the upper panel, right? So just make the connection for readers.

8) Figures S3 and S9: the most informative part of this figure is the local resolution for nascent chains. Perhaps indicate where it starts and tRNA ends.

9) Figures S5 and S10: it looks like tRNA is not shown in a relative size to the ribosome, which is a bit confusing. Its better if the size is consistent for all the components throughput the panels of the same figure. The standard ribosomal defining features need to be labeled on the LSU and SSU for orientation of readers.

10) Figure S6: this figure shows ribosomal components and how they interact with the nascent chain. First, it needs to be open about the quality of the density for the nascent chain. For the ribosome, the quality is good, and what is important is the flexibility. Therefore, I think this figure can be designed better to illustrate the important points. For example, the ribosomal components can be shown as a model colored by B-factor, which would illustrate their relative flexibility. Then, the nascent chain can be shown with local resolution to add creditability to the modeliing. Finally, it would help to have the labels in all the panels please, so that readers can follow specific changes.

11) Figure S11: please use the same representation, so that differences can stand out and label the residues in both cases. It is a bit confusing that the rest of the 50S is shown for only one case. Will also help to indicate in the inset the relevant region.

Referee #3:

The manuscript by Agirrezabala describes original results of a study addressing the concerted mRNA translation and nascent protein folding inside the peptide tunnel. The authors employ a number of biophysical techniques including circular dichroism spectroscopy, in vitro translation kinetics, PET-FCS and very notably, cryo-EM. The authors demonstrate quite elegantly how a beta barrel protein (composed of 5 beta strands) commences folding inside the nascent peptide tunnel as a flexible alpha helix. Once the nascent peptide (through its N-terminal end) reaches the end of the tunnel, it refolds to a beta hairpin that remains flexible till it releases from the ribosome.
These findings represent a corner stone in the field of cotranslational folding, and mRNA translation more broadly. While completely unexpected, the described results open new perspectives in the regulation of the ribosome activity through nascent protein folding.

The overwhelming quantity and quality of the described analysis are commendable and reflect the depth in the design of the experiments reported by the manuscript. The manuscript is well written, and the figures are clear. All the supp. figures are all justified and report important details of the performed analysis.

There are no major concerns about the validity of the reported findings.

I only have minor suggestions concerning some of the cryo-EM figures and panels:

The density threshold of the map segments displayed in figures 2, 3 and 5C is a bit low in my opinion, which makes it a bit difficult to appreciate the local resolution directly from the displayed panels (although very thoroughly reported in the supp. figures). I suggest raising the threshold to show the nice agreement between the bulky side chains/bases and density maps. The authors might want to consider mesh representation over solid transparent surfaces, but this is a purely aesthetic suggestion.

*** As a service to authors, The EMBO Journal offers the possibility to directly transfer declined manuscripts to another EMBO Press title (EMBO Reports, EMBO Molecular Medicine, Molecular Systems Biology) or to the open access journal Life Science Alliance launched in partnership between EMBO Press, Rockefeller University Press and Cold Spring Harbor Laboratory Press. The full manuscript (including reviewer comments, where applicable and if chosen) will be automatically forwarded to the receiving journal, to allow for fast handling and a prompt decision on your manuscript. For more details of this service, and to transfer your manuscript to another EMBO title please follow this link:
Link Not Available

2nd Sep 2021

Re: EMBOJ-2021-109175R-Q
Switching from α-helical to β-strand conformation upon cotranslational protein folding

Dear Marina,

Thank you for again for the comments and clarifications regarding our recent decision on your manuscript EMBOJ-2021-109175. In light of this and additional referee comments, we would like to now invite you to prepare and submit a revised manuscript. As discussed, it will be crucial to fully address all points that referees #2 and #3 raise. This includes ensuring that all data acquired is accessible to the referees and public in the event of final publication. In addition, it will also be important to revise the manuscript to discuss and clarify the concerns referee #1 has brought up. In particular, the current findings should be further discussed in the context of previous work and the specific points in which they advance the understanding contrasted to prior results. Please also include a detailed point-by-point response to each of the referees' comments when submitting your revised manuscript.

Please note that it is our policy to allow only a single round of major revision. Acceptance depends on a positive outcome of a second round of review and therefore on the completeness of your responses included in the next, final version of the manuscript. We realize that lab work worldwide may currently still be affected by the COVID-19/SARS-CoV-2 pandemic and that an experimental revision can be delayed. We can extend the revision time when needed, and we have extended our 'scooping protection policy' to cover the period required for a full revision. However, it is nonetheless important to clarify any questions and concerns at this stage. Please contact us as soon as possible if any major delays become foreseeable.

Thank you again for the opportunity to consider your work for publication. I look forward to receiving your revised manuscript.

Kind regards,

Stefanie

Stefanie Boehm
Editor
The EMBO Journal

-

Referee #1 (Report for Author)

The manuscript by Agirrezabala et al. investigates the conformations of short nascent chains of the cold shock protein CspA within the exit tunnel of the ribosome. Although the native structure of CspA is a small 5-strand beta-barrel, the authors show, using a combination of experimental techniques, that the CspA N-terminus adopts an alpha-helical conformation while 'high' in the tunnel (close to the peptidyl transferase center (PTC)). Once the nascent chain is sufficiently long as to reach the wider 'vestibule' portion near the end of the nascent chain exit tunnel, there is a conformational switch to a more native-like beta-hairpin conformation. Overall, this study, which combines a tour de force of kinetic PET measurements, cryoEM, force-profile measurements and other biochemical and biophysical analyses, represents a solid contribution to our still very sparse understanding of co-translational protein folding mechanisms. The experiments are well-constructed and controlled. However, previous studies from other groups, including von Heijne and Beckmann using force-profile and cryoEM ((2015 Cell Reports) and (2016) FEBS Lett.), and Johnson and Bernstein using FRET ((2004) Cell and (2006) Mol. Cell), have reported similar folding behavior for short nascent chains within the ribosome exit tunnel. For this reason, I expect the new insights from this study will be primarily of interest to others in this immediate research area (protein synthesis and co-translational folding), rather than a broader audience.

Reply: We thank the referee for describing our work as experimental 'tour de force' and stating that the experiments are well-constructed and controlled. We politely disagree, however, that previous studies have reported similar folding behavior. Specifically, none of the papers cited by the referee presents a folding path of a β-structured protein as it is synthesized by the ribosome. The main message of our paper, as stated in the title and the abstract, is that a β-structured protein initially folds into an α-helix inside the ribosome exit tunnel and rearranges into its native fold only upon emerging from the ribosome. This is the first time such folding pathway is shown, in particular, in combination between cryo-EM – which shows not only the type of fold but also interactions with the ribosome – and biophysical methods which indicate that folding occurs co-translationally in real-time. Generally, although it is known that β-structured protein can fold cotranslationally, the current understanding is that they are mostly unstructured inside the ribosome exit tunnel and start to fold when fully emerged into the cytosol, as we describe in the Introduction, p. 2. Information about the dynamics of β-structured nascent peptides inside the ribosome, which is reported in our paper using PET approach, is missing completely.

Concerning the papers brought up by the referee, Johnson and Bernstein examined cotranslational folding of a transmembrane helix of a membrane protein and found that it can compact inside the ribosome exit tunnel; there was no cryo-EM structure and no co-translational real-time studies. Those studies have no overlap with our work except for sharing the (established) view that protein folding can start cotranslationally. Gunnar von Heijne has developed the FPA method, which is one of several methods used in our paper, but they did not apply any of the time resolved methods to study cotranslational folding that we use in our study. Neither von Heijne nor Beckmann labs reported that a β-stranded protein formed an α-helix inside the ribosome or any real-time cotranslational folding studies. Furthermore, there are very few examples of cryo-EM structures of nascent peptides on the ribosome, unless they have a specialized stalling sequence (for detailed review, see Fig. 3 in https://doi.org/10.3390/biom10010097).

In line with the referee's comments, we have revised the text to show how our data advance the understanding contrasted to prior results (pp. 2, 6-7).

I have only one major concern with this study, which is the authors' claim that "nascent polypeptide folding could locally regulate the elongation rate by modulating aa-tRNA accommodation" (final paragraph of main text) and "These data suggest a link between the nascent peptide conformation and the PTC activity, which may result in local attenuation of translation speed depending on the nascent chain folding" (Discussion). This is an intriguing claim but is unfortunately not supported by the results of this study, as it appears to be based only on an altered position for A2602 in the cryoEM structure of nascent CspA-27 at low contour level. Making such a mechanistic claim would require, at a minimum, demonstrating that the A2602 position is sensitive to the foldability of CspA-27, and that this A2602 position does affect PTC activity.

Reply: We agree with the referee that it would be desirable to have experimental evidence to demonstrate that a particular conformation of A2602 results in the reduction of the rate of peptide bond formation/peptidyl-tRNA hydrolysis. This is, however, clearly not feasible, as this would require measurement of the catalytic activity on a fraction of ribosomes that carry that particular nascent chain conformation, which is not doable by any of the techniques available so far. However, we can link the orientation of A2602 with the nascent peptide conformation, as this is what the cryo-EM structures show. We note that it is not unusual to interpret densities at different contour levels, and there are many examples of this in cryo-EM literature (e.g. see PMID: 30089917, PMID: 32612237, PMID: 32687489, PMID: 31768042, PMID: 32924932 for examples from different groups).

Concerning the link between the orientation of A2602 and its role in catalysis, it is known from the structural and biochemical studies that the position and orientation of nucleotides at the catalytic core of the ribosome are crucial (as shown by structures from the Tom Steitz lab). A2602 is one of the key players, in particular due to its mobility (numerous papers by Ada Yonath, a pertinent reference is now added on p. 7). However, our argument is much simpler: movement of A2602 towards the A site might result in a steric clash with aa-tRNA, which is expected to slow down the accommodation and thereby affects the pace of translation. To address the referee's comment, we revised the sentences on pp. 6 (marked) and 7: 'This network extends towards the PTC and might favor a particular orientation of A2602 or of the L27 N-terminus, key residues that modulate the accessibility of the PTC for aminoacyl-tRNA or release factor binding (Baram & Yonath, 2005), thereby attenuating translation via a two-way crosstalk.' We note that the contour level for A2602 is not 'low' as stated by the referee but only somewhat lower compared to the rest of the structure. We included the exact information on the contour level on p. 6.

One minor point:
The abstract uses "configuration" to describe the differences in nascent chain conformation (not configuration).

Reply: Revised as suggested, thank you.

Referee #2 (Report for Author)

This study investigates folding of the beta-sheet protein CspA inside of the ribosomal exit tunnel. Nascent peptides typically interact with the tunnel walls, and formation of α-helices inside the tunnel has been reported for several proteins, including both types: soluble and membrane. This is explained by stability considerations, as α-helix is the most stable secondary structure. The current study illustrates that the same principle is also applied to a beta-sheet protein.

The study first shows three cryo-EM structures with a short CspA version, where the resolution for the nascent chain is 4-6 Å, allowing secondary structure determination, but not permitting residue tracing. The differences between the structures appear to be in the density inside the ribosomal tunnel, which is difficult to classify due to a disordered nature of the nascent peptide, and therefore all three classes probably represent a mixture of similar but slightly different partial folding. Interestingly, the comparison between the states shows changes in the orientation of positively charged Arg residues of proteins uL4 and uL22.

Next, previously established force profile approach was used to map the appearance of folding events by employing SecM stalling sequence. Specific folding events were detected, and the results were confirmed by photoinduced electron transfer method.

Reply: We note that the photoinduced electron transfer (PET) experiments are not merely confirmatory. Rather, they show <u>cotranslational folding events in real time</u>, thereby providing important information that cannot be extracted from the FPA measurements. The unique feature of our work is that it combines time-resolved cotranslational folding experiments with the analysis of RNCs captured at key points in translation.

The manuscript then describes cryo-EM analysis of the full CspA length. Here two different classes are reported. Although the local resolution is not indicated, again they probably represent a mixture of partially folded nascent chains, and in one of the classes the nascent chain density is not continuous. While it is not explained why two classes were selected, and not any other number, the authors could identify somewhat distinct conformations of some of the ribosomal residues, although as mentioned in the text, a lower threshold of the map was needed for some cases due to the flexibility and mixed conformations.

Reply: The local resolution of CspA70 structures is shown in Appendix Fig S9. Multiple additional runs with different combinations for class number, regularization parameter T (tau_fudge) and masks were run (not shown in Appendix Fig S8; see also replies to specific questions below). We selected these two final classes because the nascent chain signal for CspA70-1 is continuous for the entire segment, from C-term to N-term, and of enough quality to be interpreted. Signal for CspA70-2 nascent chain is not continuous, but this in fact is informative when considered together with the L27-2602 contact, interaction not reported in the literature so far. This contact is not seen in CspA70-1 and L27 is more disordered, as is in most ribosome structures. Thus, this interaction might have relevant functional implications during termination, that is, the peptide is not released unless certain conformation is sensed by the ribosome.

A lower threshold was necessary to visualize A2602 in the CspA-27 complexes due to its flexibility (we note that the maps contour thresholds are still at around 1.5σ in all three cases. This information is now given in the main text p. 6, as well as Appendix Fig. S12, legend). The rest of the PTC nucleotides, residues lining the tunnel wall, as well as other ribosome parts and tRNAs are

better resolved, and are displayed throughout the figures using higher contour levels (2σ or higher). The L27-2602 contact observed in CspA70-2 can be visualized when using a counter level around 1.7σ. This information is now given in Fig. 5 legend as well.

The organisation of the manuscript is logical and convenient to follow, the authors also clearly describe the limitations of the study in the cryo-EM part, which is obviously a weak point of this work.

I have some technical comments and suggestions regarding the presentation of the study that the authors might want considering to improve the communication of their work.

Cryo-EM part:

1) The classification of the full CspA length, shown in Figure S8 would benefit from some clarification. A particular concern is that 2 classes contain similar number of particles (53%/47%), and therefore an additional indication is needed. Please describe the entire workflow and how the result was obtained, particularly what was the data when classification into 3 and 4 classes was performed. It is particularly important because for the short CspA version, a different classification into 3 classes was performed.

Reply: The classification workflow for both CspA27 and CspA70 was first intended to isolate the subset of ribosomes containing $tRNA^{Ser}$ and $tRNA^{Leu}$, respectively, at the P-site. For that, we used a soft-edged mask that accounted for the variable arm of these tRNAs. This strategy provided a straightforward way to isolate ribosome complexes that were unequivocally in-register (i.e., contain the correct tRNA at the P-site and a vacant A site), as this variable arm is only present in $tRNA^{Ser}$, $tRNA^{Leu}$ and $tRNA^{Tyr}$, which in in the CspA sequence appear at codons 2, 27, 35, 44, 52, 57, and 69 ($tRNA^{Ser}$), 45 and 70 ($tRNA^{Leu}$) and 42 ($tRNA^{Tyr}$). Next, the workflow process was designed to isolate complexes that showed sufficient signal for the nascent chain (Methods, pp. 12, 13). This was achieved in additional multiple runs with different combinations for number of classes, regularization parameter T and/or masks that accounted for the tunnel in a hierarchical manner (at some classification runs, the discrete number of classes was set up as high as to 10); the result of this classification is shown in Fig. S3 for CspA27 and Fig. S8 for CspA70. The visual inspection of the resulting classes (mostly signal quality for nascent chain as well as other elements lining the tunnel wall and at the PTC), and the presence/absence of redundant or empty classes, in conjunction with local resolution assessments guided the entire workflow. We have improved the text to make the rationale for the classification strategy clearer (p. 13, lines 4-10).

Regarding the CspA70 classification in particular, we would like to clarify that the final split into two classes was performed to sort out heterogeneity at the PTC level (this is also clarified in the text now, see p. 13, lines 18-20). At the previous step in the hierarchy, class 1 already showed strong and continuous signal for the nascent chain, but heterogeneity at the PTC level (mostly scattered signal for L27) was detected by visual inspection. So again, different classification runs with different combinations were performed. As mentioned in a previous answer, the results that are finally presented are the ones that combined the better quality and overall local resolution for the nascent chain on one hand (CspA70-1), and the better resolved L27 in the other (CspA70-2). The resulting two classes show a similar number of particles, but are very distinct, as one shows discontinuous density for the chain and a clear A2602-L27 contact (CspA70-2), and the other (CspA70-1), continuous density for nascent chain and no contact. When classification into 3 and 4 classes was performed (we note that different numbers were also tried, combined with different regularization parameter T and/or masks), the results were suboptimal due to poorer definition for nascent chain, L27 or both in the resulting classes. The Methods section (pp. 12-13) was modified to make the process workflow clearer and avoid misunderstandings.

Related to the issue of sorting, we agree with the referee's comment that '*classes probably represent a mixture of similar but slightly different partial folding*'. This heterogeneity arises from finite number of classes that can be used for sorting. Such constraints by necessity limit the studies of biological processes, which are quasi-continuous *per se*. In principle, an infinite amount of classes would be needed to properly describe individual conformations, and given a finite data size the number of particles per class would approach zero. When a limited number of classes is used instead, each class will still contain residual structural heterogeneity as correctly pointed out. We note, however, that this is a fundamental problem of all cryo-EM studies, and does not pertain to this work alone.

As also noted by the referee, the comparison between the states shows clear changes in the orientation of certain tunnel wall components (in particular, Arg residues of proteins uL4 and uL22), as well as protein L27. The fact that these residues, which are generally well ordered and defined, show changes in orientation and differentiate among the classes indirectly conveys to the idea that the discrete classes we defined represent meaningful approximations to conformational states of the nascent chain present in the specimen.

2) A table summarizing the cryo-EM data collection and processing parameters would help.

Reply: Appendix Table S1 now also includes cryo-EM data collection and processing parameters.

Model building part:
3) I went through the model, and it is generally ok. However, since this work illustrates conformational changes of single ribosomal residues, it is particularly important to pay attention to the quality of the model. The clash scores of 7-8 unfortunately raises unnecessary doubts in the model building. It would be a simple exercise in model building to improve the clashes, and I think for this type of work, the model needs to be sound, so that the findings are technically justified. Since the authors used Phenix for refinement, to make it easier, one way to start improving the quality of the model and taking care of the clashes is to generate hydrogens for refinement.

Reply: Hydrogens were generated for refinement, and proper restraints applied throughout the whole process. Currently available refinement methods (such as Phenix, but also COOT, Rossetta or Refmac) generally enforce accurate rotamer and Ramachandran distributions and optimize fit to density. This is sometimes fulfilled at the expense of introducing minor clashes. Also, the presence of disordered and/or flexible regions contribute to unfavorable scores. The MD force field-based refinements do guarantee lower steric clashes, but often at the expense of the other metrics (see PMID: 30829573 for a nice discussion on this topic). The clash scores for ribosome structures modeled using Coot, Phenix, etc are generally in the range from 4 to 10 (see PDB 7O19, 7N2C or 7OJ0 for recent examples from different groups). Recent structures released by the PDB can also be found reporting values in the 15-20 range (e.g., PDB 7ABZ, 7AC7, 7ACJ, 7ACR), or even higher (e.g., PDB 7A0R). The highest-resolution ribosome structure published to date, at 2Å resolution (PMID: 32924932) reports clash score values above 7 as well (PDB 7K00)**.** As reported by the Molprobity server, our clash scores all are in the 80th percentile or higher. For example, CspA27-2 Molprobity score, which combines all the geometric scores into a single value to suggest quality, is 1.69 and falls into the 90th percentile (N=27675, 0Å - 99Å). We thus think that our models are sound, even more so given that the nascent peptide density is interpreted in secondary structure terms only.

Figures:
Generally, the quality of the figures can be better. Although I understand that the authors try to keep minimal annotations, sometimes it is really difficult to follow. Here are some specific examples:

4) Figure 1: please extend the labeling to all the panels, so that it is easier to identify specific residues and compare between the states.

Reply: Additional labels were introduced.

5) Figure 2: also here, please add labels.

Reply: Labeling was extended to all panels as suggested.

6) Figure 4: please show panels A and B in the same representation, so that it is easier to compare. If I understand correctly, panel C is close-up of A, then it would be good to connect and show they are related.

Reply: Panels A and B are now shown in the same representation, as suggested. Former Panel C is now part of A to avoid misunderstandings. Labeling was extended to panel B.

7) Figure 5: please add labels, indicate in the figure what is shown, as well as the color scheme. It would make much easier to understand. Lower panel is close-up of the upper panel, right? So just make the connection for readers.

Reply: Labels were added as suggested. The color scheme for comparison between various structures is now shown graphically in A (a similar caption was added to Appendix Fig S12 as well). The color code is now also consistent throughout all panels. The lower panel in A is now connected to the upper panel, indicating that the lower panel is a close-up of the upper panel.

8) Figures S3 and S9: the most informative part of this figure is the local resolution for nascent chains. Perhaps indicate where it starts and tRNA ends.

Reply: Appendix Figures S4 and S9 now indicate where the nascent chain starts. The landmarks of ribosome structure are also labeled.

9) Figures S5 and S10: it looks like tRNA is not shown in a relative size to the ribosome, which is a bit confusing. Its better if the size is consistent for all the components throughput the panels of the same figure. The standard ribosomal defining features need to be labeled on the LSU and SSU for orientation of readers.

Reply: the size of the tRNA was changed for consistency, as suggested. The ribosome landmarks are also added.

10) Figure S6: this figure shows ribosomal components and how they interact with the nascent chain. First, it needs to be open about the quality of the density for the nascent chain. For the ribosome, the quality is good, and what is important is the flexibility. Therefore, I think this figure can be designed better to illustrate the important points. For example, the ribosomal components can be shown as a model colored by B-factor, which would illustrate their relative flexibility. Then, the nascent chain can be shown with local resolution to add creditability to the modeliing. Finally, it would help to have the labels in all the panels please, so that readers can follow specific changes.

Reply: Additional captions have been included to Appendix Figure S6 as suggested. The captions to the right now show the tunnel wall components colored by B-factor and the local resolution for each of the nascent chains. Labels are now shown in panels B and C as well.

11) Figure S11: please use the same representation, so that differences can stand out and label the residues in both cases. It is a bit confusing that the rest of the 50S is shown for only one case. Will also help to indicate in the inset the relevant region.

Reply: Panels A and B are shown in the same representation as suggested. Labeling was also extended to panel B. Showing the rest of the 50S subunit would hide all of the interactions in the tunnel and is not the intention of the Fig.

Referee #3 (Report for Author)

The manuscript by Agirrezabala describes original results of a study addressing the concerted mRNA translation and nascent protein folding inside the peptide tunnel. The authors employ a number of biophysical techniques including circular dichroism spectroscopy, in vitro translation kinetics, PET-FCS and very notably, cryo-EM. The authors demonstrate quite elegantly how a beta barrel protein (composed of 5 beta strands) commences folding inside the nascent peptide tunnel as a flexible alpha helix. Once the nascent peptide (through its N-terminal end) reaches the end of the tunnel, it refolds to a beta hairpin that remains flexible till it releases from the ribosome.
These findings represent a corner stone in the field of cotranslational folding, and mRNA translation more broadly. While completely unexpected, the described results open new perspectives in the regulation of the ribosome activity through nascent protein folding.

The overwhelming quantity and quality of the described analysis are commendable and reflect the depth in the design of the experiments reported by the manuscript. The manuscript is well written, and the figures are clear. All the supp. figures are all justified and report important details of the performed analysis.

Reply: We thank the referee for her/his positive comments.

There are no major concerns about the validity of the reported findings.
I only have minor suggestions concerning some of the cryo-EM figures and panels:

The density threshold of the map segments displayed in figures 2, 3 and 5C is a bit low in my opinion, which makes it a bit difficult to appreciate the local resolution directly from the displayed panels (although very thoroughly reported in the supp. figures). I suggest raising the threshold to show the nice agreement between the bulky side chains/bases and density maps. The authors might want to consider mesh representation over solid transparent surfaces, but this is a purely aesthetic suggestion.

Reply: The contour levels for the maps have been adjusted to keep the density for the nascent chain continuous for the entire segment from C-term to N-term. The maps contour thresholds are set at around 2σ in all cases, consistent in all figures and movies. As shown in Appendix Figures S4 and S9, there is local resolution variability along the nascent chains. Some regions are better resolved and thus can be displayed at higher thresholds; other nascent chain parts on the other hand must be displayed at lower threshold as they are even more flexible and the overall signal is weaker, in particular for the cryoEM density of many side chains.

22nd Nov 2021

Re: EMBOJ-2021-109175R1
Switching from α-helical to β-strand conformation upon cotranslational protein folding

Dear Marina,

Thank you for submitting the revised version of your manuscript. Please excuse the delay in communicating this decision to you, which was due to delayed referee responses, as well as absences from the office. We have now received the reports from two of the initial referees (see comments below) and I am pleased to say that they overall find that their comments have been satisfactorily addressed and now support publication. Referee #2 has a remaining suggestion regarding information on resolution, which can be added to the final manuscript. In this version, I would also ask you to address a number of editorial issues that are listed in detail below. Please add all final edits using the "track changes" option in the manuscript file the data editors have added their notes to (please see below). Once these remaining issues are resolved, we will be happy to formally accept the manuscript for publication.

Thank you again for giving us the chance to consider your manuscript for The EMBO Journal. I look forward to receiving your final revision. Please feel free to contact me if you have further questions regarding the revision or any of the specific points listed below.

Kind regards,

Stefanie

Stefanie Boehm
Editor
The EMBO Journal

Referee #2:

The authors addressed the comments and improved the quality of the presentation of their work.
I think it would be beneficial to also indicate in the main text what is a local resolution when specific regions are described.

Referee #3:

The authors have replied appropriately to my comments. While this reviewer had only one minor comment, and thus is easy to satisfy, reviewers 1 and 2 have raised more substantial criticism and comments. It is my opinion that the authors have responded to reviewers' 1 and 2, inherently within the limitations of the current manuscript.

Regarding the possible regulatory role of nascent peptides folding in modulation the elongation rate through the positioning of A2602 (raised by reviewer 1), I believe that the authors did present compelling arguments in favor of their view, in addition of rewording their concluding statement adequately.

Reviewer 2 didn't raise major concerns and the authors did reply satisfactorily to all her/his concerns, at least in my opinion.

Finally, I would like to stress out that this work, likewise any other, presents limitations that are inherent to the applied methodologies and when the Editor(s) offer(s) the opportunity to revise the manuscript, it is implied that the authors will remain within the range of the scope of their work. Therefore, I see no reason to request any further experiments before accepting the manuscript Agirrezabala et al.

The authors have made all requested editorial changes.

Re: EMBOJ-2021-109175R2
A switch from α-helical to β-strand conformation during co-translational protein folding

Thank you again for submitting the final revised version of your manuscript. I am pleased to inform you that we have now accepted it for publication in The EMBO Journal.

EMBO PRESS

YOU MUST COMPLETE ALL CELLS WITH A PINK BACKGROUND ➔

PLEASE NOTE THAT THIS CHECKLIST WILL BE PUBLISHED ALONGSIDE YOUR PAPER

Corresponding Author Name: Marina Rodnina, Xabier Agirrezabala
Journal Submitted to: EMBO J
Manuscript Number: EMBO J-2021-109175R-Q

USEFUL LINKS FOR COMPLETING THIS FORM

http://www.antibodypedia.com
http://1degreebio.org
http://www.equator-network.org/reporting-guidelines/improving-bioscience-research-report

http://grants.nih.gov/grants/olaw/olaw.htm
http://www.mrc.ac.uk/Ourresearch/Ethicsresearchguidance/Useofanimals/index.htm
http://ClinicalTrials.gov
http://www.consort-statement.org
http://www.consort-statement.org/checklists/view/32-consort/66-title
http://www.equator-network.org/reporting-guidelines/reporting-recommendations-for-tumo
http://datadryad.org
http://figshare.com
http://www.ncbi.nlm.nih.gov/gap
http://www.ebi.ac.uk/ega
http://biomodels.net/
http://biomodels.net/miriam/
http://jjj.biochem.sun.ac.za
https://osp.od.nih.gov/biosafety-biosecurity-and-emerging-biotechnology/
http://www.selectagents.gov/

Reporting Checklist For Life Sciences Articles (Rev. June 2017)

This checklist is used to ensure good reporting standards and to improve the reproducibility of published results. These guidelines are consistent with the Principles and Guidelines for Reporting Preclinical Research issued by the NIH in 2014. Please follow the journal's authorship guidelines in preparing your manuscript.

A- Figures
1. Data
The data shown in figures should satisfy the following conditions:

- the data were obtained and processed according to the field's best practice and are presented to reflect the results of the experiments in an accurate and unbiased manner.
- figure panels include only data points, measurements or observations that can be compared to each other in a scientifically meaningful way.
- graphs include clearly labeled error bars for independent experiments and sample sizes. Unless justified, error bars should not be shown for technical replicates.
- if n< 5, the individual data points from each experiment should be plotted and any statistical test employed should be justified
- Source Data should be included to report the data underlying graphs. Please follow the guidelines set out in the author ship guidelines on Data Presentation.

2. Captions

Each figure caption should contain the following information, for each panel where they are relevant:

- a specification of the experimental system investigated (eg cell line, species name).
- the assay(s) and method(s) used to carry out the reported observations and measurements
- an explicit mention of the biological and chemical entity(ies) that are being measured.
- an explicit mention of the biological and chemical entity(ies) that are altered/varied/perturbed in a controlled manner.

- the exact sample size (n) for each experimental group/condition, given as a number, not a range;
- a description of the sample collection allowing the reader to understand whether the samples represent technical or biological replicates (including how many animals, litters, cultures, etc.).
- a statement of how many times the experiment shown was independently replicated in the laboratory.
- definitions of statistical methods and measures:
  - common tests, such as t-test (please specify whether paired vs. unpaired), simple χ2 tests, Wilcoxon and Mann-Whitney tests, can be unambiguously identified by name only, but more complex techniques should be described in the methods section;
  - are tests one-sided or two-sided?
  - are there adjustments for multiple comparisons?
  - exact statistical test results, e.g., P values = x but not P values < x;
  - definition of 'center values' as median or average;
  - definition of error bars as s.d. or s.e.m.

Any descriptions too long for the figure legend should be included in the methods section and/or with the source data.

In the pink boxes below, please ensure that the answers to the following questions are reported in the manuscript itself. Every question should be answered. If the question is not relevant to your research, please write NA (non applicable). We encourage you to include a specific subsection in the methods section for statistics, reagents, animal models and human subjects.

B- Statistics and general methods

Please fill out these boxes ➔ (Do not worry if you cannot see all your text once you press return)

1.a. How was the sample size chosen to ensure adequate power to detect a pre-specified effect size?	not applicable, as no pre-specified effect size is involved
1.b. For animal studies, include a statement about sample size estimate even if no statistical methods were used.	no animal studies
2. Describe inclusion/exclusion criteria if samples or animals were excluded from the analysis. Were the criteria pre-established?	no animal studies
3. Were any steps taken to minimize the effects of subjective bias when allocating animals/samples to treatment (e.g. randomization procedure)? If yes, please describe.	no anomal studies
For animal studies, include a statement about randomization even if no randomization was used.	no animal studies
4.a. Were any steps taken to minimize the effects of subjective bias during group allocation or/and when assessing results (e.g. blinding of the investigator)? If yes please describe.	not applicable
4.b. For animal studies, include a statement about blinding even if no blinding was done	no animal studies
5. For every figure, are statistical tests justified as appropriate?	Yes. Number of replicates is indicated in every Fig. Legend.
Do the data meet the assumptions of the tests (e.g., normal distribution)? Describe any methods used to assess it.	not applicable
Is there an estimate of variation within each group of data?	Variation within each group of data is indicated by error bars in Fogs 1 and 3 and described in Fig. Legends.

Is the variance similar between the groups that are being statistically compared?	not applicable

C- Reagents

6. To show that antibodies were profiled for use in the system under study (assay and species), provide a citation, catalog number and/or clone number, supplementary information or reference to an antibody validation profile. e.g., Antibodypedia (see link list at top right), 1DegreeBio (see link list at top right).	n/a
7. Identify the source of cell lines and report if they were recently authenticated (e.g., by STR profiling) and tested for mycoplasma contamination.	n/a

* for all hyperlinks, please see the table at the top right of the document

D- Animal Models

8. Report species, strain, gender, age of animals and genetic modification status where applicable. Please detail housing and husbandry conditions and the source of animals.	not applicable
9. For experiments involving live vertebrates, include a statement of compliance with ethical regulations and identify the committee(s) approving the experiments.	not applicable
10. We recommend consulting the ARRIVE guidelines (see link list at top right) (PLoS Biol. 8(6), e1000412, 2010) to ensure that other relevant aspects of animal studies are adequately reported. See author guidelines, under 'Reporting Guidelines'. See also: NIH (see link list at top right) and MRC (see link list at top right) recommendations. Please confirm compliance.	not applicable

E- Human Subjects

11. Identify the committee(s) approving the study protocol.	n/a
12. Include a statement confirming that informed consent was obtained from all subjects and that the experiments conformed to the principles set out in the WMA Declaration of Helsinki and the Department of Health and Human Services Belmont Report.	n/a
13. For publication of patient photos, include a statement confirming that consent to publish was obtained.	n/a
14. Report any restrictions on the availability (and/or on the use) of human data or samples.	n/a
15. Report the clinical trial registration number (at ClinicalTrials.gov or equivalent), where applicable.	n/a
16. For phase II and III randomized controlled trials, please refer to the CONSORT flow diagram (see link list at top right) and submit the CONSORT checklist (see link list at top right) with your submission. See author guidelines, under 'Reporting Guidelines'. Please confirm you have submitted this list.	n/a
17. For tumor marker prognostic studies, we recommend that you follow the REMARK reporting guidelines (see link list at top right). See author guidelines, under 'Reporting Guidelines'. Please confirm you have followed these guidelines.	n/a

F- Data Accessibility

18: Provide a "Data Availability" section at the end of the Materials & Methods, listing the accession codes for data generated in this study and deposited in a public database (e.g. RNA-Seq data: Gene Expression Omnibus GSE39462, Proteomics data: PRIDE PXD000208 etc.) Please refer to our author guidelines for 'Data Deposition'.<br><br>Data deposition in a public repository is mandatory for:<br>a. Protein, DNA and RNA sequences<br>b. Macromolecular structures<br>c. Crystallographic data for small molecules<br>d. Functional genomics data<br>e. Proteomics and molecular interactions	The cryo-EM maps and atomic models are deposited under accession codes EMD-12636, 12928 and 12929, and PDB-7NWW, 7OIF and 7OIG (CspA27), and codes EMD-13055 and 12930, and PDB-7OT5 and 7OII (CspA70), respectively.
19. Deposition is strongly recommended for any datasets that are central and integral to the study; please consider the journal's data policy. If no structured public repository exists for a given data type, we encourage the provision of datasets in the manuscript as a Supplementary Document (see author guidelines under 'Expanded View' or in unstructured repositories such as Dryad (see link list at top right) or Figshare (see link list at top right).	n/a
20. Access to human clinical and genomic datasets should be provided with as few restrictions as possible while respecting ethical obligations to the patients and relevant medical and legal issues. If practically possible and compatible with the individual consent agreement used in the study, such data should be deposited in one of the major public access-controlled repositories such as dbGAP (see link list at top right) or EGA (see link list at top right).	n/a
21. Computational models that are central and integral to a study should be shared without restrictions and provided in a machine-readable form. The relevant accession numbers or links should be provided. When possible, standardized format (SBML, CellML) should be used instead of scripts (e.g. MATLAB). Authors are strongly encouraged to follow the MIRIAM guidelines (see link list at top right) and deposit their model in a public database such as Biomodels (see link list at top right) or JWS Online (see link list at top right). If computer source code is provided with the paper, it should be deposited in a public repository or included in supplementary information.	n/a

G- Dual use research of concern

22. Could your study fall under dual use research restrictions? Please check biosecurity documents (see link list at top right) and list of select agents and toxins (APHIS/CDC) (see link list at top right). According to our biosecurity guidelines, provide a statement only if it could.	n/a
